# Optimized Monothiol Thioredoxin Derivative (ORP100S) Protects In Vitro and In Vivo from Radiation and Chemotoxicity Without Promoting Tumor Proliferation

**DOI:** 10.1002/advs.202504426

**Published:** 2025-09-11

**Authors:** Jian Wu, Xiaobei Wang, Parker Mathews, Shaima Jabbar, Min Zhang, Haim Moskowitz, Wei Duan, David P. Nichols, George William Schaaf, John D. Olson, Andrew N. Macintyre, J. Daniel Bourland, Ivan Spasojevic, Jen‐Tsan Ashley Chi, Joel Ross, Nelson Chao, J. Mark Cline, Peter B. Heifetz, Yubin Kang

**Affiliations:** ^1^ Division of Hematologic Malignancies and Cellular Therapy Department of Medicine Duke University Medical Center Durham NC 27710 USA; ^2^ School of Medicine Duke University Durham NC 27710 USA; ^3^ OrPro Therapeutics, Inc San Diego CA 92130 USA; ^4^ Department of Pediatrics National Jewish Health Denver CO 80206 USA; ^5^ Wake Forest School of Medicine Winston‐Salem NC 27157 USA; ^6^ Duke Human Vaccine Institute DUMC Box 103020 Duke University Durham NC 27710 USA; ^7^ Division of Oncology Department of Medicine Duke University Medical Center Durham NC 277710 USA; ^8^ PK/PD Core Laboratory Duke Cancer Institute Durham NC 27710 USA; ^9^ Department of Molecular Genetics and Microbiology School of Medicine Duke University Durham NC 27710 USA

**Keywords:** cell protection, chemoprotection, ferroptosis, hematopoietic stem cells, radiation mitigation, thioredoxin

## Abstract

Human thioredoxin‐1 (TRX) is a target‐selective disulfide reductase with antioxidant, anti‐inflammatory, and regulatory functions that mitigates cellular stresses in various organ systems, providing a compelling rationale for therapeutic use as a broad‐spectrum cell protectant. However, clinical application of recombinant TRX (rhTRX) is constrained by rapid clearance and proliferative intracellular activity. To overcome these limitations, a rationally designed TRX variant, ORP100S, was engineered for enhanced stability, prolonged extracellular target engagement, and improved protective function, with development of novel single‐turnover insulin reduction and hybrid‐immunocapture LC‐MS assays. ORP100S demonstrates high‐yield expression in *E. coli* (16 g L^−1^) and exhibits significant in vivo mitigating effects when administered subcutaneously to rodents and non‐human primates exposed to otherwise‐lethal total‐body ionizing radiation. Compared to native TRX, ORP100S displays improved pharmacokinetic and pharmacodynamic properties without promoting murine or human cancer cell proliferation. Additionally, ORP100S protects hematopoietic stem/progenitor cells (HSPCs) from chemotherapy‐induced toxicity in vitro and in vivo synergistically with co‐administered granulocyte‐macrophage colony‐stimulating factor (GM‐CSF). Mechanistic studies revealed that ORP100S modulates the Kruppel‐like factor 4 (KLF4)‐p53 pathway to selectively inhibit ferroptosis in HSPCs but not cancer cells. These findings highlight the potential of ORP100S as a novel therapeutic agent for mitigating acute radiation injury and improving the safety and efficacy of chemotherapy without compromising antitumor activity.

## Graphical abstract

Native thioredoxin‐1 (TRX) stimulates proliferation and rescues stem cells and cancer cells from multiple stressors by suppressing p53 and inhibiting ferroptosis via GPX4/SLC7A11 upregulation mediated by enhanced KLF4 expression and p53 promoter binding. In contrast, engineered monothiol TRX ORP100S inhibits p53 and ferroptosis selectively in non‐cancer stem cells, mitigating off‐target cell death without promoting cancer chemo/radiation resistance.

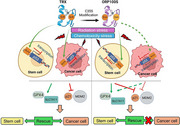

## 1 Introduction

Thioredoxins (TRXs) comprise a family of proteins involved in reduction‐oxidation (redox) reactions and are essential for life in virtually all organisms. TRX acts potently and selectively on a limited range of protein substrates to reduce intramolecular and intermolecular disulfide bonds via a two‐stage thiol – disulfide exchange mechanism (Figure S1, Supporting Information).^[^ ^1^ ^]^ TRX readily crosses cell membranes via a non‐canonical secretion/uptake mechanism^[^ ^2^ ^]^ and is a potent mitigator of stresses including effects of reactive oxygen species (ROS).^[^ ^3^ ^]^ In addition to its classical redox function, intracellular TRX acts as a growth factor and influences diverse cellular processes in both redox‐dependent and redox‐independent manners.^[^ ^4^ ^]^ The TRX system is required for the donation of electrons to ribonucleotide reductase, among other key functions, making it essential for cell survival.^[^ ^4^, ^5^ ^]^ The extracellular (secreted) functions of TRX in contrast are primarily homeostatic/protective via redox switch^[^ ^6^ ^]^ and chemokine‐like signaling^[^ ^7^ ^]^ mechanisms, and include maintenance of mucociliary transport;^[^ ^8^ ^]^ activation of mucosal peroxidases and other potent redox‐regulated anti‐oxidant proteins; and discrete anti‐inflammatory functions.^[^ ^9^, ^10^, ^11^ ^]^ TRX thus protects cells (including stem cells) from multiple exogenous and endogenous stresses and is one of the few recombinant human proteins shown to extend basal lifespan in transgenic animals.^[^ ^12^, ^13^ ^]^ Recombinant human TRX (rhTRX) has demonstrated in vitro human and in vivo animal efficacy in a wide spectrum of acute and chronic disease and injury models ranging from dermal inflammation^[^ ^14^ ^]^ to traumatic/ischemic brain injury.^[^ ^15^ ^]^

Ionizing radiation (IR) is widely applied in disease diagnosis and cancer treatment, is utilized industrially in manufacturing and power generation, and is an unavoidable hazard of air and space travel. Catastrophic IR exposure also figures prominently in the morbidity and mortality that would result from the use of nuclear weapons or radiological terrorism. Multiple organs are affected by IR depending on dose, duration, and body area exposed.^[^ ^16^ ^]^ Bone marrow (BM) and hematopoietic stem/progenitor cells (HSPCs) are among the most sensitive cells/organs to radiation injury and hematopoietic syndrome remains the first therapeutic challenge following radiation injury^[^ ^17^, ^18^ ^]^ with BM failure the leading cause of radiation‐related death. The ability of TRX to mitigate these effects post‐exposure in animals, along with a lack of direct toxicity and a wealth of non‐clinical efficacy evidence, supports the potential utility of rhTRX as a novel therapeutic agent for radiation mitigation. Indeed, our previous work has shown that intravenous (IV) rhTRX administered therapeutically, 24 h subsequent to radiation exposure, rescues BALB/c and C57Bl/6 mice from a median lethal IR dose.^[^ ^19^, ^20^ ^]^

Like radiation exposure, chemotherapeutic agents cause stresses and toxicities to stem cells. Chemotherapy is a double‐edged sword in the arsenal against cancer. While potentially curative for certain types of malignancies, chemotherapy also damages HSPCs and suppresses BM function leading to potentially fatal neutropenia, anemia, thrombocytopenia, infection, and bleeding.^[^ ^21^, ^22^ ^]^ Erythropoietin, thrombopoietin, and GM‐CSF or G‐CSF (granulocyte‐colony stimulating factor) have been used as protective agents in patients receiving chemotherapy, but with only limited efficacy.^[^ ^23^ ^]^ Moreover, all three growth factors are lineage‐specific and hence unable to promote recovery of multiple hematopoietic cell types. The recently FDA‐approved trilaciclib (Cosela), a cyclin‐dependent kinase 4/6 inhibitor, is restricted to extensive‐stage lung cancer,^[^ ^24^ ^]^ leaving a significant unmet medical need for novel agents that protect HSPCs without compromising chemotherapeutic efficacy.

Despite compelling rationale for broad efficacy, rhTRX has not yet advanced to human clinical studies. TRX in its native form has poor pharmacology^[^ ^25^ ^]^ resulting in rapid clearance, limited stability, and a very short plasma half‐life (∼30‐60 min)^[^ ^26^ ^]^ and is subject to downregulation by the selective TRX inhibitor protein TXNIP^[^ ^27^ ^]^ as well as various post‐translational modifications including S‐nitrosylation.^[^ ^28^ ^]^ The dependence of TRX activation on the thioredoxin reductase (TR)/NADPH and glutathione‐glutathione reductase (GSH/GRX) reduction systems means that endogenous TRX function is largely decoupled from gene or protein expression,^[^ ^29^ ^]^ and therapeutic use of rhTRX exogenously requires delivery in the active, reduced form. Chemical pre‐reduction improves rhTRX activity,^[^ ^30^ ^]^ but thiol oxidation and subsequent formation of inactive dimers and multimers results in poor stability and has necessitated very high dosing in animal treatment models.^[^ ^31^ ^]^ Stabilizing rhTRX in the reduced form during storage as a drug product has been difficult to achieve, requiring complex^[^ ^32^ ^]^ and potentially toxic or pro‐inflammatory formulations. Moreover, systemic administration of rhTRX may cause undesired off‐target effects as proliferative functions of the intracellular TRX‐TR system have been associated with enhanced tumor growth and resistance to chemotherapy.^[^ ^33^ ^]^

To overcome these pharmacological limitations of rhTRX we engineered and optimized its amino acid sequence, manufacturing, and formulation. Novel TRX monothiol derivative ORP100S was designed to enhance extracellular protective functions and time on target, attenuate intracellular proliferative activity, be refractory to modulation and down‐regulation by nitrosylation, and greatly extend redox stability. Here, we describe the markedly improved and drug‐like pharmacokinetics (PK) and pharmacodynamics (PD) of ORP100S which was found to retain the target selectivity and stress‐protective functions of natural TRX, but unlike rhTRX showed no capacity to stimulate tumor cell proliferation or chemoresistance yet was able to mitigate lethal effects of total body irradiation in rodents and primates. We found that ORP100S conferred significantly greater protection from radiation or chemotoxicity to stem cells and HSPCs versus cancer cells from multiple human and murine cell lines derived from both hematological and solid tumors. In contrast, rhTRX uniformly protected both cancer and non‐cancer cells. Finally, potential mechanisms underlying the differential proliferative effects of ORP100S and rhTRX between HSPCs and cancer cells were identified, demonstrating a role for ferroptosis inhibition in ORP100S‐mediated cellular protection.

## 2 Results

### 2.1 Monothiol Thioredoxin Derivative (ORP100S): Molecular Engineering, Manufacturing, and Characterization

Native TRX dithiol active‐site cysteine (Cys)_32_ and Cys_35_ form a bridging disulfide when in the inactive, oxidized form (Figure S1, Supporting Information).^[^ ^1^ ^]^ Reduction of this disulfide by TR/NADPH or GSH/GRX^[^ ^34^ ^]^ activates TRX so that deprotonation of the N‐terminal active‐site Cys_32_ forms a reactive thiolate anion capable of nucleophilic attack on a structurally compatible protein disulfide.^[^ ^1^ ^]^ This initial reaction creates a transition state comprising a mixed‐disulfide linkage to one of the two Cys of the target protein disulfide,^[^ ^1^ ^]^ exposing the normally buried Cys_35_ thiol and causing its acid‐dissociation constant (pKa) to decrease from pH 9.1 to 6.1. The now‐stabilized Cys_35_ thiolate anion attacks the mixed‐disulfide,^[^ ^35^ ^]^ releasing oxidized TRX and a fully‐reduced target protein.

**Figure 1:**
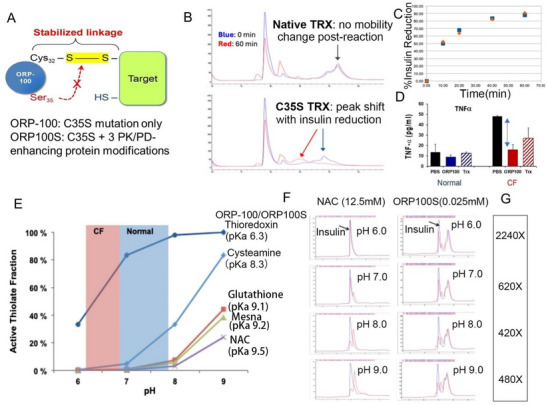
Improved properties of monothiol C35S TRX (ORP‐100 and ORP100S) versus rhTRX and thiol reducing agents. A) Codon‐optimized human TRX‐1 with mutation of the active site Cys35 to Ser (C35S TRX; ORP‐100) forms a stable mixed‐disulfide with a Cys thiol of a target protein disulfide when it reacts in the reduced state. Based on rational protein design three additional amino acid modifications were introduced in order to eliminate sites of protein:protein interactions hypothesized to negatively regulate disulfide reductase activity or redox stability (ORP100S). B) Reverse‐phase (RP)‐HPLC traces for heterodimeric human insulin at time 0 (blue) and following 60 min incubation (red) with rhTRX (top) or C35S TRX ORP‐100 (bottom). C) Relative insulin disulfide activity for rhTRX (diamonds) versus ORP‐100 (squares) calculated from change in the insulin heterodimer peak areas from 0 to 60 min determined by RP‐HPLC. D) Attenuation of inflammation in cystic fibrosis (CF) HBECs by ORP‐100. Pro‐inflammatory cytokine TNF‐alpha levels in primary human bronchial epithelial cultures from Normal (blue) and CF patient donors (red) stimulated with isotonic phosphate buffered saline (PBS; solid black bars) or PBS with 100 µm ORP‐100 (solid blue or red bars) or native TRX (hatched blue or red bars). Double arrow denotes significant difference (*P* < 0.05). E) Percentage of Cys thiols in the deprotonated, active form as a function of pH for thioredoxin/ORP‐100/ORP100S versus classical thiol agents cysteamine, glutathione, Mesna and N‐acetyl cysteine (NAC). Active thiolate fractions were calculated at each pH using the Henderson‐Haselbalch equation and published or experimentally determined (ORP‐100, ORP100S) pKa values. Blue shading: pH range of the normal human airway surface liquid (ASL) layer; pink shading: pH range of the ASL from patients with CF. F) RP‐HPLC traces for insulin reduction following 60 min incubation with either 12.5 mM NAC or 0.025 mM ORP100S (500‐fold lower concentration) at pH 6.0, 7.0, 8.0, and 9.0. Blue traces: time 0; red traces: 60 min. Overlapping red and blue traces indicate lack of reduction activity, e.g. NAC at pH 6.0. G) Relative insulin disulfide activity for ORP100S versus NAC at each pH level is shown in the boxed rectangle at right. *: *p* < 0.05; **: *p* < 0.01; ***: *p* < 0.001.

Blockade or mutation of Cys_35_ prevents mixed‐disulfide resolution, resulting in extended covalent binding to TRX targets^[^ ^36^, ^37^ ^]^ and disrupts intracellular TRX reduction by the TR/NADPH system.^[^ ^38^ ^]^ We hypothesized that these properties might improve the drug‐like characteristics of rhTRX. ORP‐100 denotes an engineered monocysteinic, chemically pre‐reduced TRX in which Cys_35_ has been replaced by redox‐inactive Ser (**Figure** **1**A).^[^ ^35^ ^]^ ORP100S has three additional non active‐site mutations, affecting multimerization and down‐regulatory post‐translational modifications, which were screened for their ability to increase stability and in vivo potency. Recombinant TRX and TRX derivatives were produced in *E. coli* by fed‐batch fermentation at 10 or 150 L scale from codon‐optimized DNA sequences using process conditions unlikely to promote amino acid misincorporation^[^ ^39^, ^40^ ^]^ and purified by anion‐exchange chromatography in the presence of the reductant dithiothreitol (DTT). Stably‐reduced drug products were manufactured by lyophilization following ultrafiltration‐diafiltration and buffer exchange to remove residual DTT. Redox stability of the lyophilized protein in the active, monomeric reduced form was attained by use of a volatile formulation. ORP100S thiol reduction state determined spectrophotometrically was found to correlate with both activity and percent monomer fraction determined either by size‐exclusion chromatography (SEC) or non‐reducing sodium dodecyl sulfate‐polyacrylamide gel electrophoresis (SDS‐PAGE) and remained in the fully active form (90% or greater reduced/monomeric) over more than four years in storage as a frozen lyophilizate. Importantly, when reconstituted in phosphate‐buffered saline (PBS) ORP100S also demonstrated markedly enhanced solution stability at 4 °C versus ORP‐100, retaining 100% activity over five days whereas ORP‐100 rapidly lost potency due to conversion to inactive dimers or multimers (data not shown).

Insulin reduction has classically been used to quantify TRX activity by absorbance change following the addition of NADPH and TR, but such catalytic coupled assays are not suitable for Cys_35_ TRX mutants which form stable insulin mixed‐disulfides stoichiometrically and cannot be redox‐cycled. We therefore utilized reverse‐phase (RP)‐HPLC to monitor directly the conversion of insulin heterodimers (containing three disulfide bonds) to monomeric or mixed‐disulfide forms following reaction with pre‐reduced native or mutant TRX over different time intervals using excess iodacetamide to stop the reactions (Figure 1B). In this single‐turnover reduction assay system the rate of decrease in insulin heterodimer peak area is dependent upon the primary nucleophilic attack by Cys_32_ regardless of whether or not the mixed‐disulfide intermediates are resolved subsequently by Cys_35_. Thus, while there were peak shifts over time with C35S TRX (Figure 1B), reflecting multiple stabilized mixed‐disulfide forms (Figure S2, Supporting Information), the rate of decrease of the large heterodimer peak for both native and mutant TRX was identical (Figure 1C). Consistent with this comparable activity both ORP100S and native TRX were found to have similarly acidic (pH 6.3) thiol pKAs (data not shown), demonstrating that the engineered modifications did not interfere with Cys_32_ thiolate anion stabilization, a key driver of the greater potency and specificity of TRX as a protein disulfide reductase versus classical thiol agents. Both ORP100S and rhTRX were found to maintain a broader pH range (Figure 1E) and many‐fold higher activity for protein disulfide reduction (Figure 1F,G) as compared to small‐molecule reducing agents like GSH and N‐acetyl cysteine (NAC).

While native rhTRX and C35S mutants could thus reduce target protein disulfides biochemically with similar kinetics, we were concerned that biological activity might be compromised because the mutants were no longer capable of redox cycling. Surprisingly however, we found that Cys_35_ mutant TRX showed greater activity in vitro and *ex vivo* versus native rhTRX. In air‐liquid interface (ALI) cultures of primary human bronchial epithelia derived from normal donors or cystic fibrosis (CF) patients, ORP‐100 attenuated saline‐stimulated release of pro‐inflammatory cytokines tumor necrosis factor alpha (TNF‐α; Figure 1D) and interleukin‐6 (IL‐6; not shown) to a significantly greater extent than did rhTRX. Increased potency versus native rhTRX was also observed for viscoelasticity normalization of CF airway mucus by ORP‐100 or ORP100S.^[^ ^41^ ^]^ These results demonstrate that the persistence of the covalent ORP100S mixed‐disulfide greatly slows reversion of the target‐protein disulfide as compared to the transient reduction by native TRX, resulting in both greater potency and longer duration of activity.

### 2.2 ORP100S is More Effective than rhTRX in Rescuing EML Hematopoietic Stem Cells from Radiation, and Unlike Native TRX, Does Not Protect or Support the Proliferation of Cancer Cells

**Figure 2:**
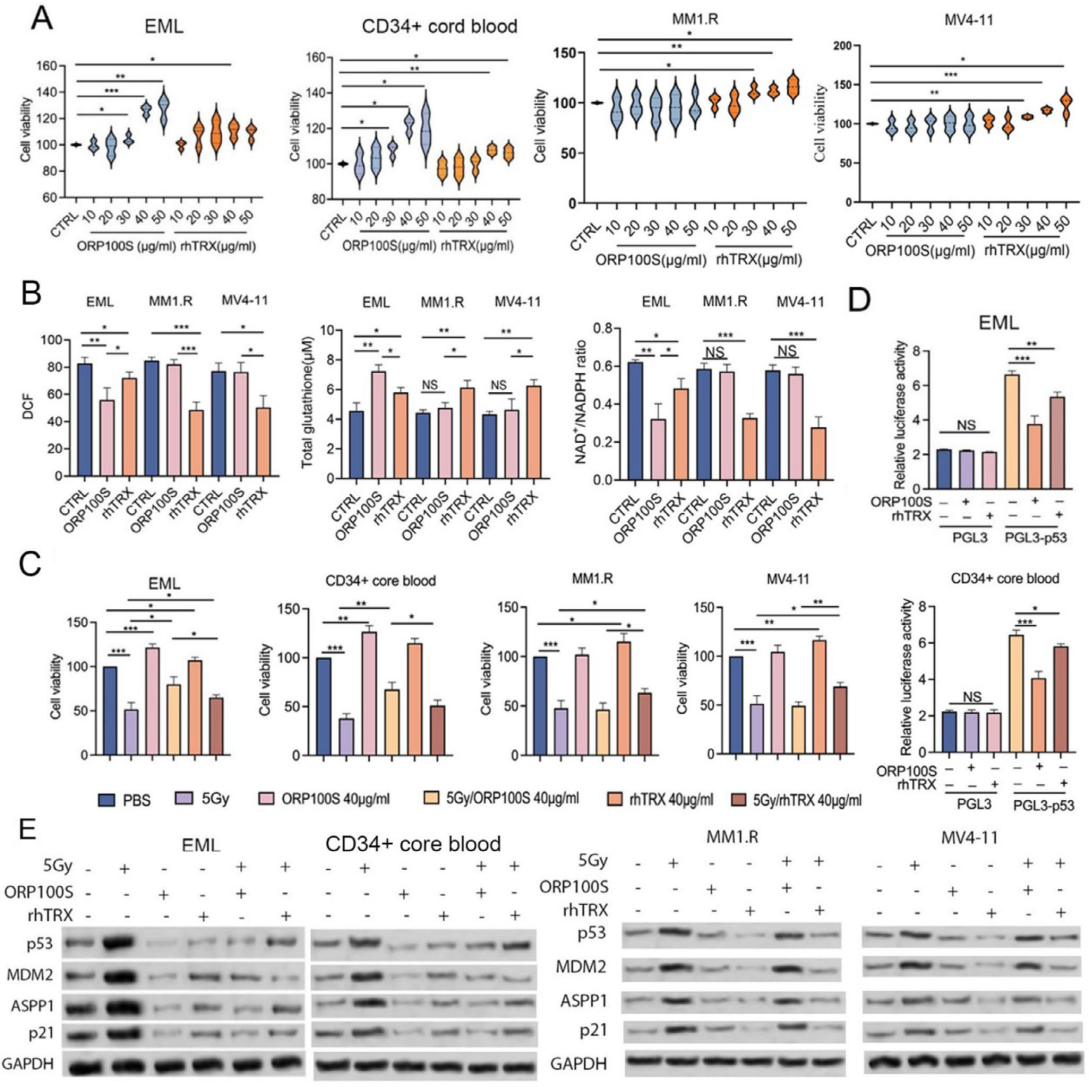
ORP100S is effective in rescuing EML cells from radiation and exhibits differential effects on EML cells versus cancer cells. A) EML cells, human cord blood CD34+ cells, MM1.R myeloma cells, and MV4‐11 leukemia cells were treated with various concentrations of ORP100S or recombinant human TRX (rhTRX) for 48 h and cell viability (MTT) was measured. Cell viability treated with PBS control buffer was set as 100%. B) EML, MM1.R, and MV4‐11 cell lines were treated with ORP100S or rhTRX in PBS (40 µg mL^−1^) for 48 h and intracellular ROS (DCF), NAD+/NADPH, and total GSH were measured. C) EML cells, human cord blood CD34+ cells, MM1.R, and MV4‐11 were irradiated (5 Gy) and treated with PBS buffer, ORP100S in PBS (40 µg mL^−1^) or rhTRX (40 µg mL^−1^) in PBS for 48 h. Cell viabilities were determined by MTS assay. Cell viability treated with PBS control buffer was set as 100%. D) For p53 transcription assay, the p53 promoter region (−1600 to −100) was cloned into a pGL3 firefly/renilla luciferase (Luc) reporter system and transduced into EML cells and human cord blood CD34+ cells. Relative Luc activity (Luc; fold change) was calculated from the ratio of p53‐pGL3 Luc activity after normalization to renilla. E) Protein lysates from samples as described in C were subjected to western blotting using the indicated antibodies. *: *p* < 0.05, **: *p* < 0.01; ***: *p* < 0.001.

Murine EML (erythroid, myeloid and lymphoid) is a BM‐derived multipotent hematopoietic cell line used to model HSPCs in vitro.^[^ ^42^ ^]^ ORP100S was found to be more effective than rhTRX in promoting the proliferation of both EML cells and CD34‐positive human cord blood cells (CD34+)^[^ ^43^ ^]^ but unlike rhTRX did not stimulate the proliferation of either hematological or solid tumor‐derived cancer cell lines (HT29 human colon cancer, MM1.R human myeloma, MV4‐11 human leukemia, B16‐F10 murine melanoma, EG7 murine lymphoma, and TRAMP murine prostate cancer) (**Figure** **2**A; Figure S3A, Supporting Information). ORP100S was also more effective than rhTRX in lowering intracellular ROS and NAD+/NADPH ratio and increasing cellular GSH in EML cells but had no effect on these stress defenses in cancer cells (Figure 2B; Figure S3B, Supporting Information). Consistent with these observations, ORP100S also protected EML cells and human cord blood CD34+ cells but not cancer cell lines from 5 Gy irradiation in vitro (Figure 2C; Figure S3C, Supporting Information) and suppressed p53 transcription in EML cells and human cord blood CD34+ cells but not cancer cells (Figure 2D; Figure S4, Supporting Information) resulting in lower levels of p53 and the p53‐regulated proteins MDM2, ASPP1, and p21 in irradiated EML cells and human cord blood CD34+ cells but not in any irradiated cancer cell lines (Figure 2E; Figure S3D, Supporting Information). In contrast, rhTRX was uniformly protective/proliferative in all cells. These data show that ORP100S exerts differential proliferative and protective effects on murine and human stem cells versus murine and human cancer cells.

### 2.3 ORP100S and rhTRX Demonstrate Differential Chemoprotective Effects in Murine Hematopoietic Stem Cells Versus Cancer Cells In Vitro

**Figure 3:**
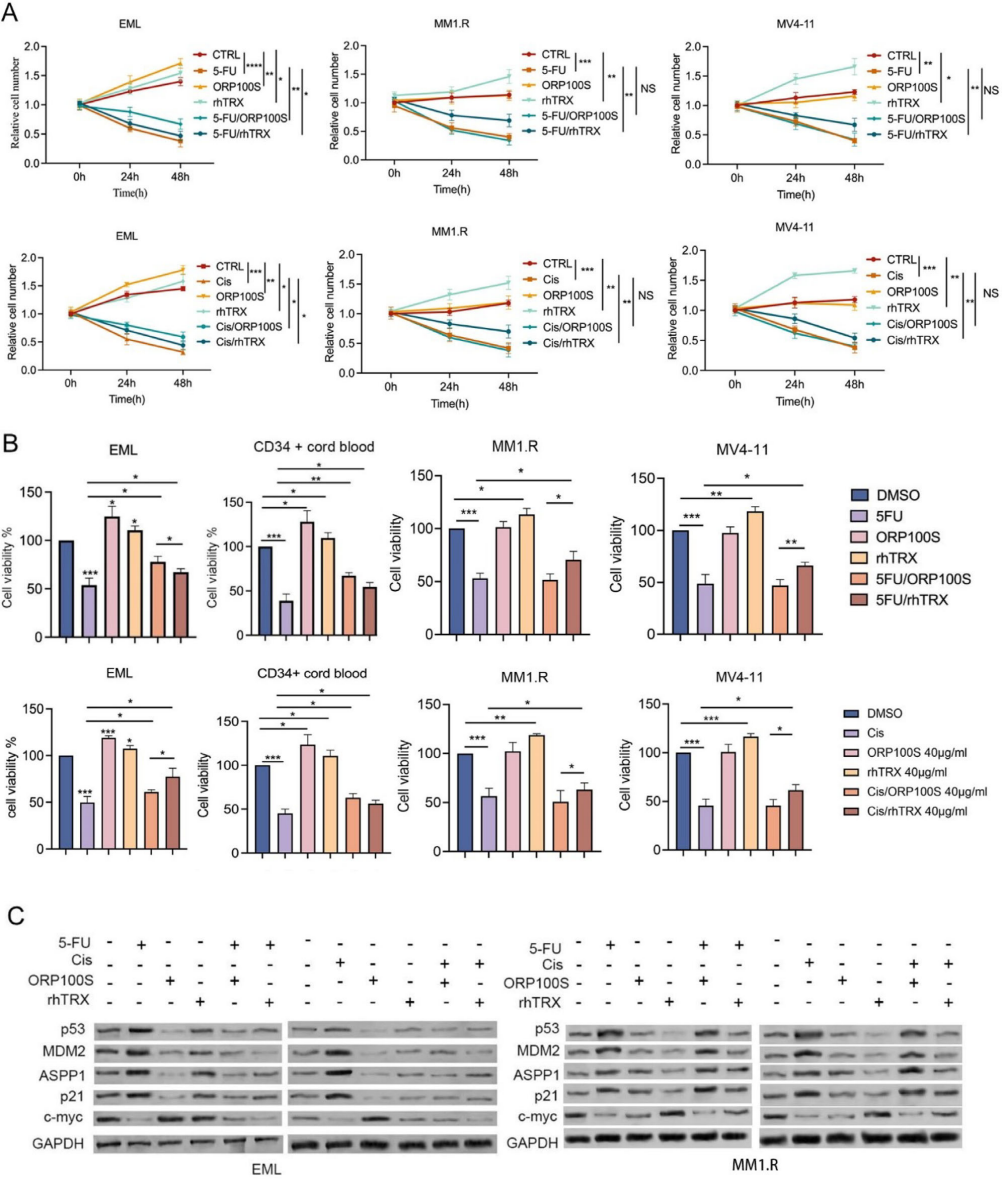
ORP100S is effective in rescuing EML and human CD34+ cells from chemotoxicity and exhibits differential effects on EML versus cancer cells. A) EML, MM1.R, and MV4‐11 cell lines were treated with 5‐FU (25 µm, upper panel) or cisplatin (1 µm, lower panel) with/without ORP100S or rhTRX (40 µg mL^−1^) for 48 h. Cell counts were determined by Trypan blue dye. Data represent the mean ± SD of three experiments. Cell number at baseline before treatment was normalized as 1. B) EML, human CD34+ HPSCs, MM1.R, and MV4‐11 cells were treated with 5‐FU (25 µm, upper panel) or cisplatin (1 µm, lower panel) with/without ORP100S or rhTRX (40 µg mL^−1^) for 48 hr. Cell viability was measured by MTT assay. Data represent mean ± SD of three experiments. Cell viability at baseline before treatment was set as 100%. C) EML cells (left panel) and MM1.R cells (right panel) were treated with 5‐FU (25 µM) or cisplatin (1 µM) with/without ORP100S or rhTRX (40 µg mL^−1^) for 48 h. Cells were lysed to obtain total protein of each group. Protein lysates were subjected to western blot analysis with indicated antibodies. *: *p* < 0.05, **: *p*,0.01; ***: *p* < 0.001.

We further tested and compared the chemoprotective and proliferative effects of ORP100S and rhTRX in response to four different chemotherapeutic agents (5‐fluorouracil, 5‐FU; cisplatin; doxorubicin; and etoposide) on mouse EML and human cord blood CD34+ stem cells and human cancer cell lines MM1.R, MV4‐11, and HT29, and murine cancer cell lines EG7, B16‐F10, and TRAMP. Cell proliferative/stimulatory effects were quantified by changes in cell number (**Figure** **3**A) as well as by thiazolyl blue tetrazolium bromide (MTT) cell viability assay (Figure 3B; Figure S5, Supporting Information). ORP100S was found to be more effective than rhTRX in rescuing murine and human stem cells from chemotoxicity, but unlike rhTRX, ORP100S was unable to protect cancer cells from chemotherapy‐mediated killing. Additionally, ORP100S attenuated chemotherapy‐mediated upregulation of the p53 pathway in EML cells but not in cancer cells (Figure 3C; Figure S6, Supporting Information).

### 2.4 ORP100S Administered 24 h Post‐Exposure Rescues Mice from a Lethal Total Body (TB) Radiation Dose and Mitigates Radiation Injury in Cynomolgus Macaques

**Figure 4:**
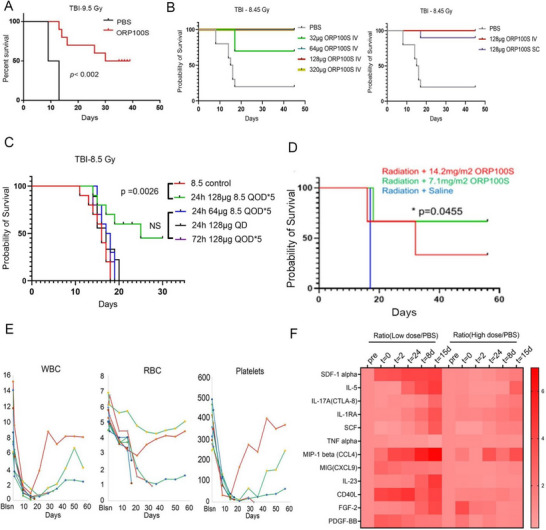
ORP100S mitigates radiation injury in mice and in cynomolgus macaques. A) C57Bl/6 mice were exposed to gamma TBI (9.5 Gy, TB) and 24 h later were administered PBS buffer or ORP100S in PBS (320 µg, IV, every other day for five doses) and survival was measured. n = 10 mice per treatment group, 5 males and 5 females. B) C57BL/6 mice received 8.45 Gy TBI and 24 h later were administered PBS buffer or ORP100S (32, 64, 128 or 320 µg, IV, 128 µg, SC) in PBS every other day for five doses. n = 10 mice per treatment group, 5 male and 5 females. C) C57BL/6 mice received 8.5 Gy TBI and were administered PBS or ORP100S in PBS (64 or 128 µg at 24 h post‐exposure QOD for five doses; 128 µg given at 24 h once; or 128 µg given at 72 h QOD for five doses). n = 10 mice per treatment group, 5 males and 5 females. Mice were monitored twice daily for weight loss and clinical symptoms and were euthanized upon reaching the humane endpoints. Overall survival was calculated from Kaplan‐Meier curves and log‐rank analysis performed from the first day of ORP100S injection until death or reaching humane endpoint. D) Female cynomolgus macaques were irradiated (single‐fraction whole body 4 Gy dose) using 6 MV X‐rays at a dose rate of 0.69 Gy min^−1^ with a Varian 2100 EX dual energy linear accelerator. Twenty‐four hours later, animals were given PBS (n = 2), ORP100S (14.2 mg m^−2^, equivalent to 128 µg per mouse, n = 3), or ORP100S (7.1 mg m^−2^, equivalent to 64 µg per mouse, n = 3) SC every other day for a total of 5 doses. Some non‐human primates had to be euthanized due to uncontrolled menses, reaching humane endpoints. These animals were humanely euthanized not due to classical hematopoietic syndrome failure but due to this unexpected issue. We treated these cases as censored data points in Kaplan‐Meier survival curves. Survival following was evaluated using Kaplan‐Meier curves and log‐rank analysis from the first day of ORP100S injection until death or reaching humane endpoints. E) Blood samples were collected before radiation, at day 1, 3, 5, 7, 9, 16, 23, 30, 37, 44, 51, and 58 post radiation. White blood cell count (WBC), hemoglobin (Hb), red blood cell count (RBC), platelets (PLT), neutrophils, lymphocytes, and monocytes were measured by cell counter. F) Cytokines and chemokines from blood samples taken at the indicated time points were measured by Thermo Fisher ProcartaPlex™ NHP 37‐plex cytokine/chemokine/growth factor panel (cat. no. EPX370‐40045‐901). The data shown are the ratios of levels between low dose/PBS‐treated and high dose/PBS‐treated. *: *p* < 0.05, **: *p* < 0.01; ***: *p* < 0.001.

We investigated the effects of ORP100S in mitigating radiation injury in rodents and nonhuman primates (NHPs). C57Bl/6 mice were irradiated at 9.5 Gray (Gy), TB, TBI and 24 h later received IV tail‐vein injection of PBS vehicle control or 320 µg (ca. 16 mg kg^−1^) ORP100S in PBS every other day (QOD) for a total of five doses. While all control animals receiving PBS alone died within 14 days, half of the mice receiving ORP100S survived through termination of the study at day 40 (**Figure** **4**A). We further performed a dose‐ranging efficacy study (32, 64, 128, 320 µg IV) using the same design with the addition of a 128 µg subcutaneous (SC) arm. Nine of ten 128 µg SC ORP100S‐treated mice and all treatment groups given ORP100S IV at greater than 64 µg survived to the end of study on day 45 (Figure 4B; Figure S7, Supporting Information). SC injections are readily administered with assurance of dose accuracy even outside of clinical settings, making SC the preferred route of administration in the event of radiation exposure incidents where large populations may require treatment in the field. Hence, we performed additional studies to establish the optimal SC dosing of ORP100S (Figure 4C). SC ORP100S at 128 µg QOD starting at 24 h post‐exposure was found to be the most effective regimen for rescuing mice from lethal TB irradiation (TBI).

We next examined if ORP100S could rescue large animals from radiation injury. Eight cynomolgus macaques were irradiated (single‐fraction whole body 4 Gy dose) using 6 MV X‐rays at a dose rate of 0.69 Gy min^−1^ with a Varian 2100 EX dual energy linear accelerator.^[^ ^44^ ^]^ Twenty‐four hours later, animals were given PBS (n = 2), ORP100S (14.2 mg m^−2^, equivalent by allometric scaling to the 128 µg per mouse dose, n = 3), or ORP100S (7.1 mg m^−2^, equivalent to 64 µg in mouse, n = 3) SC every other day for a total of five doses. ORP100S administered at 7.1 mg m^−2^ SC QOD prolonged survival significantly (p = 0.0455; Figure 4D). Following TBI, white blood cells (WBC), red blood cells (RBC), platelets (PLT), neutrophils (Neut), lymphocytes (Lymph), and monocytes (Mono) decreased through day 15 (Figure 4E; not shown) with the greatest subsequent recovery in the surviving high dose animal. The 7.1 mg m^−2^ dose also resulted in increased plasma levels of the tissue repair‐associated chemokine SDF‐1 whereas the inflammation‐associated protein CD40 ligand (CD40L) increased immediately following irradiation and decreased during the course of ORP100S exposure (Figure 4F; Figure S8, Supporting Information) consistent with expression of the CD40L partner CD40 being negatively regulated by TRX, as has been reported in non small‐cell lung cancer cells subject to TRX knock‐down.^[^ ^45^ ^]^

### 2.5 ORP100S Protects HSPCs from Chemotherapy‐Induced Hematological Toxicities Without Affecting Tumor Killing

**Figure 5:**
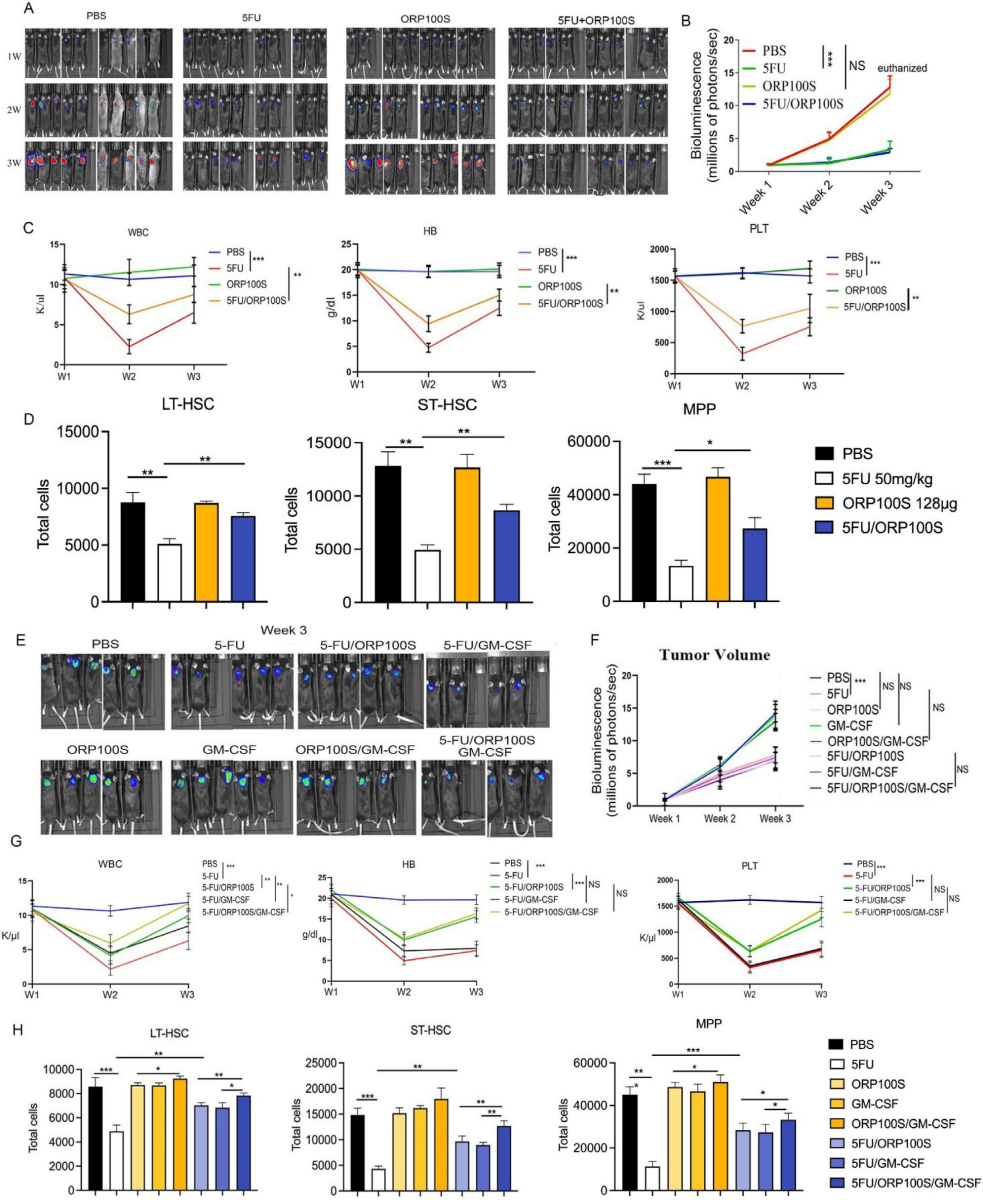
ORP100S protects hematopoietic stem cells from chemotherapy induced injury and is additive/synergistic with GM‐CSF. A–D) C57Bl/6 mice were implanted with luciferase‐expressing EG7 cells (1 × 10^6^ cells per animal), and once tumors were established, mice were treated with 5‐FU (IP, 50 mg kg^−1^, one dose) followed by PBS vehicle or ORP100S (128 µg, SC every other day for five doses). (A) Tumor volume was monitored weekly by bioluminescence. Bioluminescence intensity of individual animals from week one to week three in four groups of mice, ie., PBS (CTL), 5‐FU, ORP100S, or the combination (5‐FU and ORP100S), was measured (10 mice per group). B) Bioluminescence activity was quantified by determining the total flux (photons/sec) in each mouse from week one to week three. C) Peripheral blood was collected weekly for the measurement of white blood cell count (WBC), hemoglobin (HB) and platelet (PLT). D) Experiments were terminated at two weeks post 5‐FU injection and mice were sacrificed. Total numbers of bone marrow long‐term (LT)‐HSPC (Lin‐Scal+C‐Kit+CD150+CD48‐), short‐term (ST)‐HSPC (Lin‐Scal+C‐Kit+CD150‐CD48‐), and multi‐potential progenitor cells (MPP) (Lin‐Scal+C‐Kit+CD150‐CD48+) were measured. E–H) C57Bl/6 mice were implanted with luciferase‐expressing EG7 cells and treated subsequently with PBS or 5‐FU IP (50 mg kg^−1^, one dose). The mice were then given PBS control buffer, ORP100S (128 µg, SC every other day for five doses total), GM‐CSF (2 µg, SC, daily for five d), or a combination of ORP100S and GM‐CSF. Tumor volume was quantified weekly by measuring bioluminescence intensity and peripheral blood was drawn for hematological analysis. Mice were sacrificed at two weeks post 5‐FU injection. E) Bioluminescence imaging of tumor burden at week three for C57BL/6 mice implanted with EG7 tumor cells and treated as described (eight groups). F) Change in tumor volume over time for the indicated treatments. Bioluminescence was quantified by determining the total flux (photons/sec) in each mouse from week one to week three. G) Change in WBC, HB, and PLT in peripheral blood for the indicated groups and treatments. H) Total numbers of bone marrow LT‐HSC, ST‐HSC, and MPP cells at termination for each of the indicated treatments. Data represent mean ± SD for n = five to six mice per group. *: *p* < 0.05, **: *p*,0.01; ***: *p* < 0.001.

We next investigated the impact of ORP100S on in vivo hematopoietic recovery and tumor responses in two murine tumor chemotherapy models. Luciferase‐expressing EG7 lymphoma cells were implanted into C57Bl/6 mice (**Figure** **5**A). Once tumors were established, mice were administered 5‐FU (50 mg kg^−1^) intraperitoneally (IP) followed by SC administration of PBS vehicle or ORP100S (128 µg, every other day for five doses). 5‐FU effectively controlled tumor growth, and importantly, ORP100S neither stimulated tumor growth in the absence of 5‐FU nor attenuated 5‐FU anti‐tumor activity when co‐delivered with chemotherapy (Figure 5A,B). Treatment with ORP100S did, however, improve significantly 5‐FU induced pancytopenia, with higher levels of WBC, HB, and PLT in ORP100S‐treated mice compared to those treated with PBS vehicle (Figure 5C). Furthermore, BM long‐term (LT) HSPCs, short‐term (ST) HSPCs, and multipotent progenitors (MPPs) were increased significantly in ORP100S‐treated mice compared to PBS‐treated mice (Figure 5D). We also tested the hematopoietic protective effects of ORP100S using a B16‐F10 melanoma tumor‐cisplatin chemotherapy animal model (Figure S9A, Supporting Information). While ORP100S treatment did not interfere with efficacy of cisplatin in killing melanoma tumor cells, ORP100S reduced significantly the degree of pancytopenia, and demonstrated protection of both HSPCs and MPPs (Figure S9, Supporting Information).

Hematopoietic growth factor GM‐CSF is FDA‐approved to reduce chemotherapy‐induced neutropenic fever. In order to assess the potential for additive or synergistic effects when combined with ORP100S we compared the effectiveness of ORP100S and GM‐CSF alone or together in rescuing chemotherapy‐induced BM suppression in the EG7 tumor model (Figure 5E; Figure S10, Supporting Information). Neither ORP100S nor GM‐CSF interfered with the anti‐tumor activity of 5‐FU (Figure 5E,F), and both ORP100S and GM‐CSF showed comparable efficacy in reducing neutropenia (Figure 5G). However, while GM‐CSF had no beneficial effect on the suppression or recovery of HB and PLT, ORP100S decreased suppression and promoted their recovery (Figure 5G) and was additive to GM‐CSF in protecting BM HSPCs and MPPs (Figure 5H). ORP100S thus demonstrates additive effects with GM‐CSF for WBCs and stem/progenitor cells, but unlike GM‐CSF, is also able to protect non‐neutrophilic red cell and platelet lineages.

### 2.6 Both IV and SC ORP100S Demonstrate Drug‐Like Pharmacology and Exhibit Minimal Repeated‐Dose Toxicity in Mice

**Figure 6:**
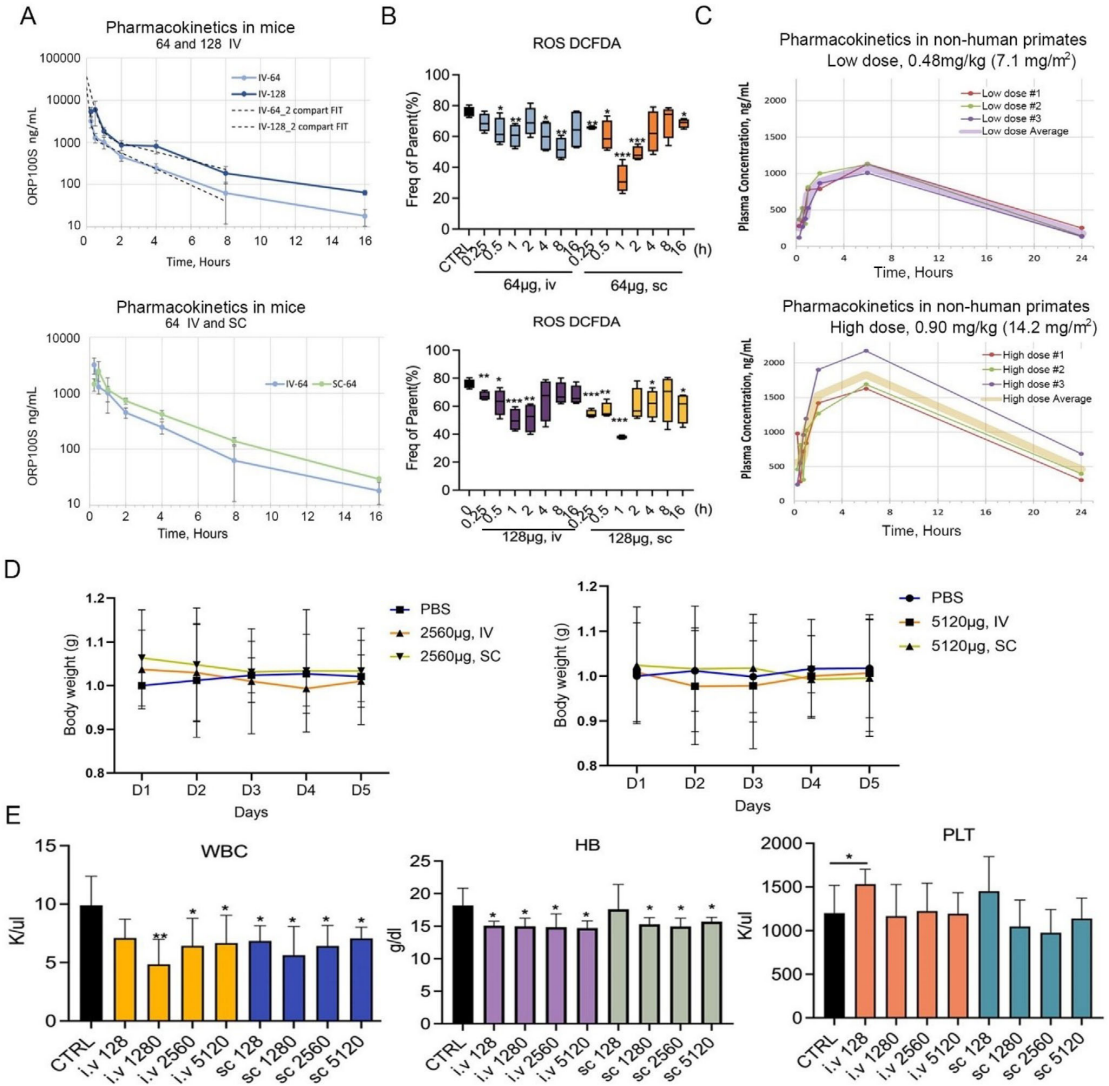
ORP100S pharmacokinetics, pharmacodynamics, and toxicology. A) PK: C57Bl/6 mice were injected iv (tail vein) or sc (dorsal) with 64 or 128 µg ORP100S in PBS. Blood samples were collected at 0, 0.25, 0.5, 1, 2, 4, 8, and 16 h post‐injection and the plasma fraction was separated. ORP100S levels in plasma were determined using hybrid immunocapture LC/MS‐MS (KCAS Bioanalytical & Biomarker Services) with appropriate dilution (assay linearity 1 to 2000 ng mL^−1^). PK parameters were determined using WinNonlin non‐compartmental analysis. n = 5 mice per time‐point. B) PD: mice were injected iv (tail vein) or sc (dorsal) with 64 or 128 µg ORP100S in PBS. Blood samples were collected at 0, 0.25, 0.5, 1, 2, 4, 8, and 16 h post‐injection. ROS levels in blood mononuclear cells were measured using a DCFDA assay kit. n = 5 mice per each time‐point. C) PK study in cynomolgus macaques. NHPs were injected sc ORP100S 7.1 mg m^−2^ (equivalent to 64 µg per mouse, low dose) or 14.2 mg m^−2^ (equivalent to 128 µg per mouse, high dose). Blood samples were collected at 0, 0.25, 0.5, 0.75, 1, 2, 6, and 24 h post‐injection and ORP100S levels in plasma were quantified by hybrid immunocapture LS/MS‐MS with appropriate sample dilution. D) Repeat‐dose acute toxicology study: Animals were injected with 0, 128, 1280, 2560, or 5120 µg IV or SC in a volume of 200 µl once daily for five days. Mice were weighed daily and monitored for changes in activity and behavior. Body weight was measured and presented as means ± SD. Body weights over time in 2 highest doses (2560 µg and 5120 µg) were shown. E) Mice were euthanized 1d after the final dose. Terminal blood samples were also collected at euthanasia. CBC: white blood cell count (WBC), hemoglobin (HB) and platelets were measured by cell counter. *: *p* < 0.05, **: *p* < 0.01; ***: *p* < 0.001.

PK and PD studies were performed in mice to compare IV and SC administration of 64 or 128 µg ORP100S. C35S monothiol TRX mutants in the reduced state were found to be refractory to standard bioanalysis methods, limiting the ability to perform PK analysis. Immunodetection of reduced but not oxidized ORP‐100 or ORP100S by enzyme‐linked immunosorbent assay (ELISA) was highly nonlinear in plasma or serum matrices due to TRX‐antibody interactions, whereas solvent extraction of ORP100S in these matrices preparatory to mass spectrometry resulted in marked loss of ORP100S signal due to partition into insoluble fractions (data not shown). To address these limitations, a sensitive hybrid‐immunocapture liquid chromatography / tandem mass spectrometry (LC/MS‐MS) bioanalytical assay with linearity from 1 to 2000 ng mL^−1^ was developed (Kansas City Analytical Services, Olathe, KS) for quantification by mass spectrometry of the ORP100S tryptic peptide SMPTFQFFK following immunoprecipitation of ORP100S from plasma. Serum half‐life t_1/2_ for SC ORP100S was 3–4 h with 100% bioavailability (**Figure** **6**A; Table S1, Supporting Information) versus 1 h reported for rhTRX even with constant infusion.^[^ ^46^ ^]^ In PD studies, IV and SC ORP100S treatment lowered ROS in blood mononuclear cells, with the maximal effect observed at 1 h post‐SC administration (Figure 6B), ca. 30 min after PK Tmax. SC administration of ORP100S had a marginally superior PD profile versus IV administration. PK in NHP differed from mouse, with slower absorption (longer Tmax, lower Cmax) and slower elimination resulting in a calculated t_1/2_ for SC ORP100S in NHP of 7 to 9 h (Figure 6C; Tables S2 and S3, Supporting Information).

We performed a repeated‐dose acute toxicology study of ORP100S in C57Bl/6 mice. Animals were injected with 0, 128, 1280, 2560, or 5120 µg ORP100S IV or SC in a volume of 200 µl in PBS once daily for five days. Mice were weighed daily and monitored for changes in activity and behavior. Terminal blood samples were collected at euthanasia one day after the final dose. Body weights remained stable even in the 2560 and 5120 µg treatment groups, with no obvious signs of toxicity (Figure 6D). H&E‐stained sections of bone marrow (sternum), liver, spleen, kidney, and lung from ORP100S‐treated mice revealed no treatment related lesions or pathological changes compared to PBS controls. All examined organs in the ORP100S treated groups exhibited normal histological architecture and cellular morphology, with no evidence of inflammation, necrosis, or other adverse alteration, consistent with a lack of acute organ toxicity across the tested dose range (Figure S11, Supporting Information). In comparison to the control group, WBC and HB blood cell counts were decreased slightly at all doses (with no further dose response beyond 128 ug) whereas at the 128 ug ORP100S IV dose PLT counts showed a slight increase but were unchanged at other IV or SC ORP100S doses. Similar trends were observed even at the highest dosage (equivalent to 80 times the 64 µg radioprotective dose), consistent with minimal toxicity at all doses evaluated (Figure 6E).

### 2.7 ORP100S and rhTRX Differentially Affect Ferroptosis Between EML Cells and Cancer Cells

**Figure 7:**
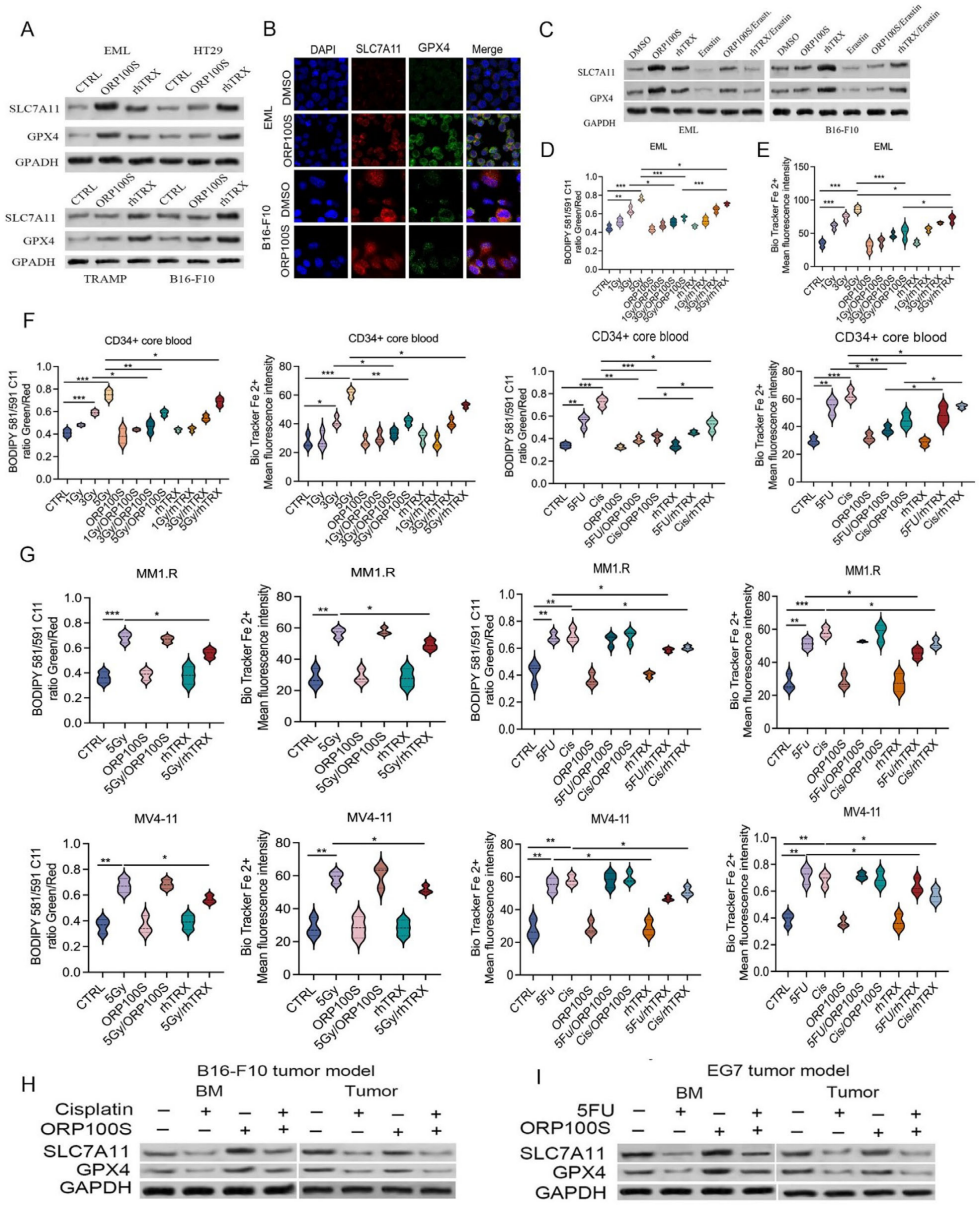
ORP100S attenuates ferroptosis induced by radiation and chemotherapy in stem cells but not in cancer cells. A) EML cells, HT29 cells, TRAMP cells, and B16‐F10 cancer cells were treated with PBS, 40 µg mL^−1^ ORP100S or 40 µg mL^−1^ rhTRX in PBS for 48 h. Cells were harvested and protein lysates were subjected to Western blotting using SLC7A11 antibody, GPX4 antibody or GAPDH antibody. B) EML cells and B16‐F10 cancer cells were treated with PBS, 40 µg mL^−1^ ORP100S or 40 µg mL^−1^ rhTRX in PBS for 48 h. The cells were transferred to pre‐coated slides and stained with immunofluorescence labeled SLC7A11 antibody, immunofluorescence labeled GPX4 antibody or 4',6‐diamidino‐2‐phenylindole (DAPI). The levels of SLC7A11 and GPX4 were detected under fluorescent microscope. Representative images were shown. C) EML cells and B16‐F10 cancer cells were treated with PBS, 40 µg mL^−1^ ORP100S or 40 µg mL^−1^ rhTRX in PBS with or without 10 µm Erastin (ferroptosis inducer) for 48 h. Cells were harvested and protein lysate was subjected to Western blot analysis with indicated antibodies. D) EML cells were irradiated with 1 Gy, 3 Gy or 5 Gy and treated with PBS, 40 µg mL^−1^ ORP100S or 40 µg mL^−1^ rhTRX in PBS for 48 h. The cells were incubated with 5 µM boron‐dipyrromethene (BODIPY) 581/911 C11 reagent in PBS at 37 °C for 30 min. Labeled cells were washed and analyzed by flow cytometry. For lipid peroxidation analysis, the peroxidation state of each group was calculated by mean fluorescence intensity (MFI) ratio of the FL1 channel (590 nm) to that of FL3 channel(510 nm). E) EML cells were irradiated with 1 Gy, 3 Gy or 5 Gy and treated with PBS, 40 µg mL^−1^ ORP100S or 40 µg mL^−1^ rhTRX in PBS for 48 h. The cells were labeled with Bio Tracker Far‐red Labile Fe2+ Dye at 5 µm for 90 min in PBS at 37 °C. Intracellular ferrous iron was measured by quantifying mean fluorescence intensity using Image J. F) Human CD34+ HSPCs were irradiated with 1 Gy, 3 Gy or 5 Gy and treated with PBS, 40 µg mL^−1^ ORP100S or 40 µg mL^−1^ rhTRX in PBS for 48 h. The cells were labeled with Bio Tracker Far‐red Labile Fe2+ Dye at 5 µm for 90 min in PBS at 37 °C. Intracellular ferrous iron was measured by quantifying mean fluorescence intensity using Image J. G) MM1.R and MV4‐11 cells were treated with 5 Gy or 5‐FU (25 µm) or cisplatin (1 µM) with or without ORP100S (40 µg mL^−1^) or rhTRX (40 µg mL^−1^) for 48 hr. Lipid peroxidation (top panels) and intracellular ferrous iron level (lower panels) were measured. H) C57Bl/6 mice were implanted with EG7 and subsequently treated with 5‐FU ± ORP100S as described in Figure 5A. At 3 weeks tumors and bone marrow were collected for SLC7A11 and GPX4 western blot analysis. I) C57Bl/6 mice were implanted with B16‐F10. Following tumor development, mice were treated with cisplatin ± ORP100S as described Figure S7 (Supporting Information). Protein lysates were subjected to Western blotting with indicated antibodies. *: *p* < 0.05, **: *p* < 0.01, ***: *p* < 0.001.

Ferroptosis, an intracellular iron‐dependent form of regulated cell death caused by lipid peroxidation and iron accumulation, plays an important role in the functional maintenance of murine HSPCs^[^ ^47^ ^]^ and in the biological processes of cancer cells.^[^ ^48^, ^49^, ^50^ ^]^ HSPCs have been reported to have low rates of protein synthesis that make them selectively vulnerable to ferroptosis.^[^ ^51^ ^]^ The amino acid transporter SLC7A11 – glutathione peroxidase‐4 (GPX4) signaling axis constitutes the major cellular regulator of ferroptosis. Inactivation of GPX4 or SLC7A11 induces ferroptosis^[^ ^52^ ^]^ and TRX has been shown to inhibit ferroptosis via a mechanism involving GPX4 activation.^[^ ^52^, ^53^ ^]^ In agreement with these observations, we found that rhTRX increased expression of SLC7A11 and GPX4 in EML cells, and to an even greater degree in cancer cells (**Figure** **7**A,B). In contrast, ORP100S upregulated expression of SLC7A11 and GPX4 in EML cells significantly but had no effect in cancer cells (Figure 7A,B; Figure S12A, Supporting Information). Treatment with the ferroptosis inducer erastin (10 µM) abrogated effects of ORP100S on GPX4 and SLC7A11 (Figure 7C; Figure S12B, Supporting Information).

Radiation exposure induces dose‐dependent increases in lipid peroxidation and intracellular ferrous iron, hallmarks of ferroptosis induction.^[^ ^54^ ^]^ In irradiated EML cells and human CD34+ HSPCs ORP100S treatment was found to lower both lipid peroxidation (BODIPY 581/591 C11 lipid probe, Figure 7D) and intracellular ferrous iron (BioTracker far‐red labile Fe^2+^ dye, Figure 7E,F; Figure S11, Supporting Information). Similarly, ORP100S treatment attenuated induction of lipid peroxidation and intracellular ferrous iron by 5‐FU and cisplatin in EML cells and human CD34+ HSPCs, but had no effect in cancer cells (Figure 7F,G; Figure S13, Supporting Information). ORP100 treatment likewise suppressed chemotherapy‐induced GPX4 and SLC7A11 downregulation in vivo in BM cells but not in tumors when evaluated in an EG7‐implanted tumor model treated with 5‐FU (Figure 7H) or in a B16‐F10 tumor‐implantation model treated with cisplatin (Figure 7I). Differential inhibition of ferroptosis therefore, appears to be a common mechanism underlying the selective ORP100S protection of non‐cancerous cells from both radiation and chemotoxic stresses.

### 2.8 p53 is Required for Ferroptosis in EML and Cancer Cells and is Affected Differently by ORP100S in EML Cells and Cancer Cells

**Figure 8:**
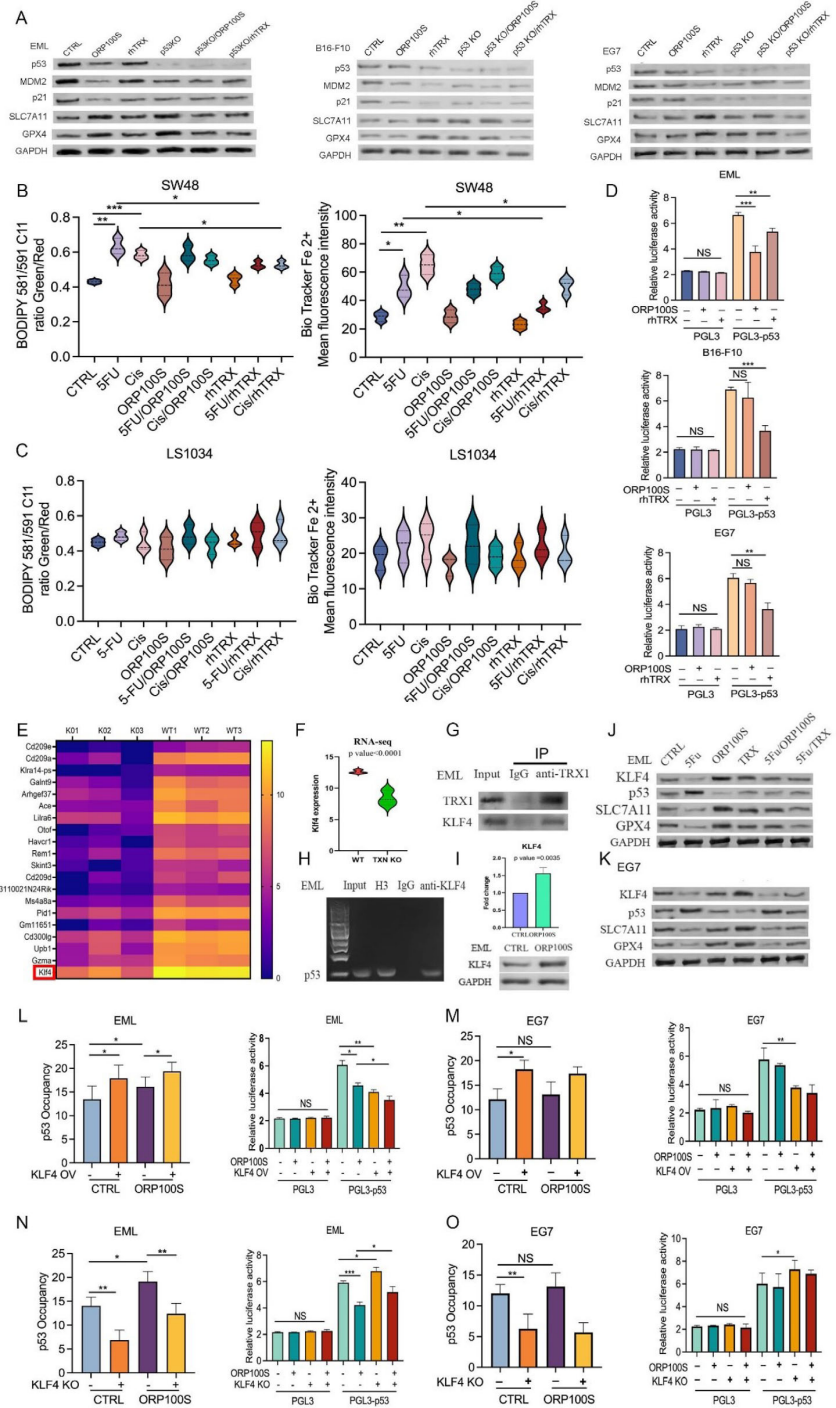
ORP100S mediates ferroptosis inhibition via the KLF4/p53 axis. (A) EML cells (left panel), B16‐F10 cancer cells (middle panel), and EG7 cancer cells (right panel) were transduced with control or p53‐specific CRISPR/Cas9 then treated with ORP100S (40 µg mL^−1^) or rhTRX (40 µg mL^−1^) and after 48 h cells were harvested, lysed and protein lysates were subjected to Western blotting with indicated antibodies. (B) SW48 cells were treated with 5‐FU (10 µM) or cisplatin (3 µM) with or without ORP100S (40 µg mL^−1^) or rhTRX (40 µg mL^−1^) for 48 hr. Cellular lipid peroxidation (left panel) and intracellular labile ferrous iron Fe^2+^ levels (lower panel) were measured by BODIPY 581/591 C11 and BioTracker Fe^2+^ fluorescence staining, respectively. C) LS1034 cells were treated with 5‐FU (5 µM) or cisplatin (10 µM) with or without ORP100S (40 µg mL^−1^) or rhTRX (40 µg mL^−1^) for 48 h and lipid peroxidation (left panel) and intracellular ferrous iron levels (right panel) were measured as described above. D) p53 promoter regions were cloned into the PGL3 firefly/renilla reporter system and the resultant PGL3‐p53‐reporter plasmids were transduced into EML cells, B16‐F10 and EG7 cell lines. Cells were treated with ORP100S (40 µg mL^−1^) or rhTRX (40 µg mL^−1^) for 48 hr, and luciferase bio‐luminescence activity was measured. E) RNA sequence heat map of enriched HSPCs from TRX knockout (KO) and WT mice (KLF4 is highlighted). F) KLF4 expression in TRX KO versus WT mice. G) Co‐immunoprecipitation (Co‐IP) of TRX with KLF4. Total cell lysates were immunoprecipitated using an anti‐TRX antibody or IgG control and the pull‐down was probed for KLF4 by immunoblotting. H) Representative images of ChIP‐PCR products amplified by primers (100 bp) on 2% agarose gel in EML cells. I) EML cells were treated with PBS or ORP100S (40 µg mL^−1^) for 48 hr. Cells were harvested and KLF4 mRNA levels were measured by qPCR (top panel). Additionally, KLF4 protein levels were measured by Western blot (lower panel). J,K) EML and EG7 cells were treated with 5‐FU (25 µM), ORP100S (40 µg mL^−1^) or TRX (40 µg mL^−1^) for 48 hr. Protein lysates were subjected to western blotting with indicated antibodies. L,M) EML cells (L) and EG7 cells (M) were transduced with a control vector or KLF4‐overexpression plasmid and then treated with PBS or 40 µg mL^−1^ ORP100S. Chromatin immunoprecipitation (ChIP)‐PCR was performed to measure the binding (occupancy) of KLF to the p53 promoter (left panel). EML and EG7 cells transduced initially with a control vector or KLF4 overexpression plasmid were subsequently transfected with a PGL3‐p53 firefly luciferase reporter vector construct and treated with ORP100S (40 µg mL^−1^), following which p53 transcription‐driven luciferase bioluminescence was measured (right panel). N,O): KLF4 in EML cells (N) and EG7 cells (O) was knocked out by CRISPR/Cas9 and cells were then treated with 40 µg mL^−1^ ORP100S. ChIP‐PCR was performed to measure the binding (occupancy) of KLF to the p53 promoter (left panel). EML and EG7 cells transduced initially with control vector or KLF4 KO plasmids were subsequently transfected with a PGL3‐p53 firefly luciferase reporter vector construct and treated with ORP100S (40 µg mL^−1^), following which p53 transcription‐driven luciferase bioluminescence was measured (right panel). Data represent means ± SD of three independent experiments *: *p* < 0.05, **: *p* < 0.01, ***: *p* < 0.001.

Activation of pathways controlled by the tumor‐suppressor protein p53 following oxidative stress or exposure to toxic chemicals is tightly associated with ferroptosis and cell death.^[^ ^55^ ^]^ Using TRX conditional knockout mice, we demonstrated previously that TRX regulates HSPCs through p53.^[^ ^56^ ^]^ In the current study, ORP100S was found to downregulate p53 and inhibit 5‐FU‐ and cisplatin‐induced p53‐MDM2‐ASPP1‐p21 signaling as well as ferroptosis in EML cells (Figure 3C), but not in cancer cells (Figure S6, Supporting Information). Knockout of p53 increased the levels of GPX4 and SLC7A11 in all cells but prevented their further upregulation by rhTRX or ORP100S (**Figure** **8**A), consistent with TRX‐mediated inhibition of ferroptosis operating via p53 suppression. To further understand the role of p53 in chemotherapy‐induced ferroptosis, human colon cancer cells with or without functional p53 mutations were compared.^[^ ^57^ ^]^ Exposure to chemotherapeutic agents induced ferroptosis in SW48 cells (p53 wildtype) but not in LS1034 cells (p53 mutant: exon 7 codon 245 G to A nucleotide substitution, resulting in Gly to Ser mutation),^[^ ^57^ ^]^ and this induction was reversible by rhTRX but not ORP100S (Figure 8B,C; Figure S14, Supporting Information). Similarly, in SW48 cells, cisplatin or 5‐FU treatment led to the stabilization of p53 and the induction of p21. These expression changes were attenuated by rhTRX but, notably, not by ORP100S. In LS1034, containing mutant p53, chemotherapy was unable to induce p21 with no further effect by either rhTRX or ORP100S on p21 or MDM2 (Figure S15, Supporting Information). These data suggest a critical role of p53 in mediating differential ferroptosis activity between EML cells and cancer cells.

To further elucidate the mechanism underlying the divergent effects of ORP100S and TRX on EML cells and cancer cells we utilized a reporter gene system for p53 transcription. The mouse p53 gene promoter/regulator region was cloned upstream of a firefly luciferase gene and transduced into either EML cells or cancer cells, which were then treated with rhTRX or ORP100S. ORP100S was found to be twice as potent as rhTRX for suppression of p53 expression in EML cells, but had no effect on p53 transcription in any of the four cancer cell lines tested (Figure 8D; Figure S16, Supporting Information). In contrast, rhTRX suppressed p53 transcription in all cells. These data suggest that TRX protects from stress‐induced ferroptosis via inhibition of p53 induction, and that the differential proliferative/protective effects of ORP100S and rhTRX may result from selective inability of ORP100S to down‐regulate expression of p53 at the transcriptional level in cancer cells.

### 2.9 ORP100S Affects KLF4, a p53 Transcription Factor, Differently Between EML Cells and Cancer Cells

Proteins in the TRX family lack DNA‐binding motifs and are not known to have innate DNA‐binding activity. Consequently, the effect of TRX on p53 transcriptional control is likely to be exerted indirectly via interaction with one or more DNA‐binding regulatory proteins. Analysis of RNA seq data obtained using HSPCs isolated from TRX‐knockout mice^[^ ^56^ ^]^ revealed that Krüppel‐like factor 4 (KLF4) was decreased significantly in TRX‐depleted HSPCs (Figure 8E,F), suggesting a possible association between the two proteins. KLF4 is a tumor suppressor that has been reported to interact physically with p53 and its coding sequence, both suppressing p53 gene transcription^[^ ^58^ ^]^ and increasing p53 DNA‐binding affinity and target selectivity.^[^ ^59^, ^60^ ^]^ Using co‐immunoprecipitation and chromatin immunoprecipitation (ChIP)‐PCR we found that TRX forms a complex with KLF4 (Figure 8G) which binds the p53 gene promoter (Figure 8H). Treatment with ORP100S upregulated KLF4 expression in EML cells but had no effect on KLF4 in EG7 cancer cells whereas rhTRX increased KLF4 several fold versus control in EG7 and to a lesser extent in EML (Figure 8I–K). To understand the function of KLF4 in the context of TRX‐mediated effects on p53 transcription in EML cells versus cancer cells, we overexpressed KLF4 in EML and EG7 cells (Figure S17, Supporting Information) and then treated with ORP100S. ORP100S enhanced KLF4 binding to the p53 promoter and increased KLF4‐mediated suppression of p53 transcription in EML (Figure 8L) but not EG7 cells (Figure 8M). In a complementary study, we knocked out KLF4 by CRISPR in EML and EG7, respectively (Figure S17, Supporting Information). In the absence of KLF4, ORP100S‐mediated p53 occupancy and p53 transcriptional inhibition were attenuated significantly in EML cells (Figure 8N), but there was no effect in EG7 cells (Figure 8O). These data demonstrate that ORP100S lacks the ability to mediate KLF4 transcriptional down‐regulation of p53 in cancer cells.

## 3 Discussion

In the current study, we present data demonstrating markedly enhanced pharmacological properties for the rationally‐designed and pharmacologically‐optimized TRX monocysteinic active site derivative ORP100S as compared to native rhTRX. We also show efficient manufacturing of a stable drug product at high yield with supporting bioanalytical and activity assays. The PK profile of ORP100S supports SC administration as single or repeated doses with significant bioavailability and exposure versus the massive intraperitoneal (IP) bolus^[^ ^31^ ^]^ or constant IV infusion^[^ ^46^ ^]^ required for effective dosing of native TRX. ORP100S showed 100% bioavailability when administered SC with greater PD response than IV injection. This is a particularly significant finding in the context of potential management of widespread acute radiation injury where large populations may be exposed and self‐administration or field treatment outside of a clinical setting may be required. Importantly, ORP100S was able to rescue irradiated HSPCs but not irradiated cancer cells, and unlike native TRX, did not support or stimulate cancer cell proliferation. The striking observation that ORP100S improved significantly survival of mice and NHPs exposed to otherwise‐lethal radiation therefore opens up new drug development avenues for mitigation of lethal radiation injury. Moreover, ORP100S exerts its cell‐protective activity via a hematological mechanism distinct from current FDA‐approved radiotherapeutic agents, with the potential for additive/synergistic combination as well as suppression of inflammation including attenuation of radiation‐induced CD40L increase in primates observed following ORP100S dosing. Protective pulmonary effects are also supported by our observation that C35S mutant TRX attenuates saline stress‐induced proinflammatory cytokines in primary human bronchial epithelium cultured at an air‐liquid interface.

Radiation exposure poses a significant potential threat to public health, necessitating the development of effective countermeasures. There are five FDA‐approved agents for managing hematopoietic acute radiation sickness (H‐ARS): Neupogen (G‐CSF), Neulasta (PEGylated G‐CSF), Udenyca (biosimilar to Neulasta), Leukine (GM‐CSF) and Nplate (thrombopoietin analog). The efficacy of these hematopoietic growth factors is limited, cell lineage‐specific, and primarily relevant for low‐dose exposure.^[^ ^61^ ^]^ No treatments have yet been approved for gastrointestinal or radiation‐induced lung injury. Because of the central role of ROS in driving radiation injury, general antioxidants such as NAC or GSH have been tested in the prevention or treatment of radiation injury. Even when dosed frequently (at least daily), and ideally before or within a few hours of radiation exposure, these agents have shown only marginal efficacy.^[^ ^62^ ^]^

SC administration of ORP100S in mice was also found to protect HSPCs and ameliorate chemotherapy‐induced pancytopenia without affecting the anti‐tumor efficacy of chemotherapy in vivo. Unlike lineage‐specific hematopoietic growth factors, ORP100S promoted the recovery of all hematopoietic lineages and its effects were additive with GM‐CSF, accelerating neutrophilic recovery. Hematological toxicity is one of the most common adverse events of chemotherapy, necessitating dose reduction and treatment interruption which can reduce the efficacy of a potentially curative treatment. Our current study presents initial non‐clinical efficacy data for ORP100S‐mediated chemoprotection of hematopoietic cells, and evaluates a likely mechanism. Together these suggest a compelling therapeutic role for ORP100S in mitigating chemotherapy‐associated myelotoxicities and relieving therapeutic dose‐limitation.

### 3.1 Ferroptosis and p53

A key finding of our study is that ORP100S rescues EML stem cells but not cancer cells from ferroptosis, a recently characterized cell death mechanism associated with iron accumulation, GSH depletion, lipid peroxidation formation, ROS accumulation, and down‐regulation of SLC7A11 and GPX4.^[^ ^63^ ^]^ A primary mechanism of the wildtype p53 protein is to repress expression of SLC7A11, a central component of System Xc‐, a cystine/glutamate antiporter that imports cystine into cells where it is reduced to cysteine, a precursor for GSH which in turn promotes GPX4 peroxidase activity to prevent ferroptosis via detoxification of lipid peroxides. Ferroptosis plays an important role in the functional maintenance of HSPCs^[^ ^47^ ^]^ and cancer cells, and while some cancer cells exhibit greater or lesser sensitivity to ferroptosis, it has been observed that cancer cells that are resistant to apoptosis are exquisitely vulnerable to ferroptosis.^[^ ^48^, ^50^ ^]^ The differential effect of TRX and ORP100S on cancer cells is intriguing and unlikely to be due to the initially high levels of GPX4 and SLC7A11 observed in some cancer cells and tumors. We also do not yet know if ORP100S and TRX exhibit similar differential effects on other forms of regulated cell death between EML cells and cancer cells. HSPCs and cancer cells undergo several types of regulated cell death in response to stress depending on the injurious agent and the intensity and duration of the injury, and TRX is known to play various roles in regulating the activity of transcription factors and in inhibition of ferroptosis^[^ ^53^ ^]^ as well as apoptosis.^[^ ^64^ ^]^ For example, C35S TRX mutants (or non‐redox active C32S) have been shown to decrease apoptosis under stress conditions by constitutively binding ASK‐1 and inhibiting downstream ASK‐1 induced JNK activation and caspase 3 activity^[^ ^65^ ^]^ but it is unclear if such effects would differentiate between cancerous and non‐cancerous cells as ORP100S appears to. However, the likeliest conclusion from our data is that inhibition of ferroptosis underpins the protective activity of ORP100S we observed for radiation and chemotoxic stresses, and our results strongly suggest that the differential effects on ferroptosis in hematopoietic versus cancer cells involve modulation of p53 transcription.

The p53 protein in cancer acts primarily as a tumor suppressor due to its ability to elicit cell‐cycle arrest, apoptosis and /or senescence in response to cellular stress^[^ ^66^, ^67^ ^]^ and mutations in p53 cause loss of tumor suppressor functions. P53 directly represses the expression of SLC7A11 to inhibit ferroptosis and transactivates both its own negative regulator MDM2 and the cyclin‐dependent kinase inhibitor p21, leading to cell cycle arrest. Besides transcriptional control, p53 is tightly regulated by diverse post‐translational modifications (PTMs), such as phosphorylation, acetylation, ubiquitination and others.^[^ ^68^ ^]^ p53‐mediated cellular functions are highly orchestrated via the close connection of these PTMs^[^ ^69^ ^]^ and it is hypothesized that the profile of p53 PTMs induced by specific stimuli reflect unique cellular responses.^[^ ^70^ ^]^ For example, phosphorylation of p53 at Ser_46_ was found to induce apoptosis but not cell‐cycle arrest. p53 PTMs are therefore dependent on p53‐reactivating signal type, and may also vary depending on intracellular context, such as DNA damage or oxidative stress, in specific cell types or in response to different stress signals.^[^ ^71^ ^]^ In addition, stress signals can promote both “activating” and “repressing” PTMs that can fine‐tune p53 activity in particular settings.^[^ ^72^ ^]^ Future investigations may provide further insight into the specific role of each PTM and how ORP100S and TRX may affect each PTM differently and in different cell types.

The overall role of TRX in p53‐mediated stress responses is likely complex, as TRX has been shown to modulate downstream p53 trans‐activation functions post‐transcriptionally via enhancement of sequence‐specific DNA binding, in contrast to the upstream ability of TRX but not ORP100S to down‐regulate p53 expression transcriptionally in cancer cells as demonstrated here. Since TRX in the reduced form does not interact directly with wildtype p53 even though TR activity is required for transactivation by human p53 in vitro,^[^ ^73^ ^]^ TRX‐mediated p53 transactivation control is most likely indirect, as has been shown for Ref‐1.^[^ ^74^ ^]^ Furthermore, our data demonstrate that rhTRX, but not ORP100S, attenuates the ability of chemotherapeutic agents to cause increased expression of p53 and its downstream transcriptional targets MDM2 and p21 only in p53‐wildtype cancer cells. In contrast, neither rhTRX or ORP100S were able to rescue LS1034 p53‐mutant cells, consistent with the inability of mutant p53 to transactivate MDM2 and p21 expression.^[^ ^75^ ^]^ While differential crosstalk between endogenous TRX and wildtype or mutant p53 might occur due to redox function, because C35S mutant TRX lacks intracellular redox cycling it is unlikely that ORP100S would exert differential protective effects in mutant versus wildtype p53 cancers.

### 3.2 KLF4

KLF4 is a stress‐responsive transcription factor that suppresses p53 expression in a target‐selective manner.^[^ ^59^, ^60^ ^]^ KLF4 acts directly on the p53 promoter to signal RAS(V12)‐mediated transformation and resistance to DNA‐damage‐induced apoptosis, and induction of KLF4 by the saponin polyphyllin III has recently been shown to play a role in downregulating intracellular accumulation of lipid ROS and ferroptosis.^[^ ^76^ ^]^ Similarly, our results demonstrate that differences in the ability of ORP100S to modulate activity of KLF4 in EML cells and cancer cells are associated with differential ferroptosis inhibition‐related and stress‐protective effects in response to both radiation and chemotoxicity. We hypothesize that direct or indirect protein‐protein interaction between TRX/ORP100S and KLF4 acts to enhance KLF4‐mediated transcriptional repression of p53 expression via two potential molecular mechanisms: a) stabilization of KLF4‐DNA binding activity due to direct interaction with TRX/ORP100S; and/or b) enhanced occupancy by KLF4 of the negative‐regulatory ‐972 to ‐953 p53 promoter region mediated by direct or indirect interaction with TRX/ORP100S. Either mechanism would result in lower p53 protein levels and attenuated induction of downstream p53 targets (MDM2, ASPP1, p21). It is not yet determined whether the interaction of ORP100S with KLF4 is redox dependent (thus requiring reduced ORP100S) or is mediated via non Cys‐dependent binding as is the case for TRX inhibition of ASK‐1 in the apoptotic cascade. KLF4 is a tumor suppressor with context‐dependent expression^[^ ^77^ ^]^ regulated by discrete transcription factors as well as via autoregulatory binding to its own promoter region.^[^ ^78^ ^]^ Our results show that ORP100S increased KLF4 expression in normal cells but not in cancer cells. Native TRX, on the other hand, increased KLF4 in both normal and cancer cells and also supported their proliferation. The inability of ORP100S to boost KLF4 in cancer cells could be related to the previously‐reported inability of Cys_35_ TRX mutants to stimulate proliferation in cancer cells,^[^ ^38^ ^]^ as well as differences in uptake or cancer cells’ altered signaling environment. For example, constitutive oxidative stress in cancer cells might inactivate reduced ORP100S, or other oncogenic pathways might override KLF4 control of p53. Further experiments, such as co‐immunoprecipitation assays under reducing versus non‐reducing conditions, or mutating cysteine residues on KLF4, would be required to confirm if ORP100S binding to KLF4 is direct (or indirect via additional trans‐acting factors) and whether or not it depends on ORP100S reduction state.

ORP100S and TRX may also differentially affect other p53‐associated transcription factors such as RBP‐Jk and C/EBP*b*‐2. The RBP‐Jk transcription factor binds the ‐972 to ‐953 region of the p53 gene promoter and acts as a transcriptional repressor of p53 expression^[^ ^79^ ^]^ during the entry into S‐phase. The RBP‐Jk amino acid sequence contains 13 Cys residues, and using DIPro^[^ ^80^ ^]^ (UC Irvine http://scratch.proteomics.ics.uci.edu) we identified five high‐probability disulfide bonds between Cys residue pairs 54/58, 78/86, 285/299, 389/440, and 238/272, some of which may be TRX targets. C/EBP*b*‐2 is an enhancer of p53 expression^[^ ^81^ ^]^ and targets the same region of the p53 promoter as RBP‐Jk. TRX/ORP100S may reduce these disulfides to modulate DNA binding or transcriptional regulatory activity as has been shown for other TRX‐regulated transcription factors. It is also possible that TRX/ORP100S may modulate the p53 transcriptional control system via a non‐disulfide mechanism similar to that involving ASK‐1 inhibition^[^ ^65^ ^]^ mediated by binding of TRX Trp_31_ when this residue is exposed by reduction or mutation of the TRX active‐site Cys_32_ or Cys_35_.^[^ ^64^ ^]^ Further work is needed to fully characterize differences in the interaction between ORP100S and KLF4 in EML cells and cancer cells, and whether ORP100S and TRX may also affect other p53‐associated transcription factors.

### 3.3 ORP100S Exhibits Improved Drug Properties

A significant limitation to therapeutic supplementation with recombinant TRX has been rapid clearance, restricting its effective use in animals to transgenic overexpression, extreme IP bolus dosing, or continuous infusion, none of which are suitable routes of administration for human therapeutics. In contrast, we observed the plasma half‐life of ORP100S to be several fold longer than that reported for native rhTRX. We hypothesize that prolonged covalent binding to circulatory proteins disulfides may be at least partially responsible for improved ORP100S PK. Albumin and the Fc region of certain antibodies have disulfides that are abundant targets for TRX in the bloodstream, and these would be expected to react very rapidly with reduced ORP100S. Such targets could serve as reservoirs for extracellular ORP100S which binds via mixed disulfide until released in the reduced form by thiol‐disulfide exchange with endogenous TRX or the GSH‐GRX system, acting to shield ORP100S from the rapid renal clearance reported to drive the distribution of native rhTRX from the circulation. In a similar manner exchange of ORP100S from bound peripheral blood target proteins may slow partitioning into the systemic circulation, underlying the longer Tmax and higher AUC observed for SC versus IV ORP100S administration.

While TRX is known to protect many cell types in various models, our data for ORP100S presented here is focused on the hematopoietic context. Our unpublished work has shown that ORP100S is functional in the lung versus muco‐obstructive, inflammatory and oxidative stresses, consistent with our findings in this study for ORP100S in primary bronchial epithelia and as an attenuator of the induction by radiation exposure in primates of the inflammatory regulatory CD40L. In animal and human lung models we have generally observed greater anti‐inflammatory and muco‐modulatory activity in vivo, ex vivo, and in vitro (in 3D human lung organoids) for ORP100S than native TRX.

### 3.4 ORP100S: Attenuated Intracellular Redox Function

In our studies ORP100S appears to retain the broad cytoprotective functionality of TRX for normal cells but unlike TRX does not confer protection on cancer cells. Our hypothesis is that ORP100S activity is attenuated intracellularly as a redox doner compared to TRX, and that this has a comparatively larger effect on cancer cells. The TRX/TR system is important for many cancers, and intracellular reductase TR is a well‐established target for cancer cell killing.^[^ ^82^ ^]^ Two characteristics of ORP100S support this model. First, C35S TRX mutants are known to be refractory to reduction intracellularly by the TR/NADPH system^[^ ^38^ ^]^ and second, ORP100S forms stable covalent linkages via mixed disulfide following nucleophilic attack on compatible target protein disulfide bonds. Thus, when oxidized, ORP100S forms either Cys_32_–Cys_32_ homodimers or mixed‐disulfide protein adducts and these species cannot be redox cycled back to the reduced form intracellularly by the same mechanism utilized by native TRX. Instead, ORP100S re‐reduction must occur through thiol‐disulfide exchange, either by reaction with endogenous reduced TRX, or via thiols such as the GSH‐GSH reductase (GSH‐GR) or GSH/GRX systems. The GSH/GRX system in particular is known to serve as a backup pathway to reduce oxidized thioredoxin‐1 when TR is inhibited.^[^ ^34^ ^]^ Although the TRX and GSH systems can cross‐supply electrons as a compensatory mechanism, they cannot fully substitute for each other intracellularly due to differences in substrate specificity and efficiency making intracellular TRX dependent on the NAPDH‐dependent TRX reductase system.^[^ ^83^ ^]^ However, in extracellular compartments where TRX is secreted abundantly such as the airway and other epithelial surfaces including the eye and gut, there is typically insufficient or absent TR and/or NADPH but abundant GSH/GRX. The protective, extracellular functions of TRX may therefore depend more on a functional GSH/GRX system than the largely proliferative intracellular TRX that is redox cycled by TR/NADPH.

Native TRX crosses cellular membranes, including the nuclear envelope, via a poorly‐characterized leaderless mechanism that does not require secretion/uptake signals or involve endocytosis.^[^ ^84^ ^]^ Since this mechanism is independent of redox state^[^ ^85^ ^]^ it is likely that ORP100S utilizes the same process. As cancer cells have higher levels of ROS with generally more oxidizing environments there would be more protein disulfides present to act as covalent traps for intracellular ORP100S but not TRX, limiting the amount of reduced ORP100S available to interact with KLF4. Moreover, as described below we hypothesize that any ORP100S that does enter cells will primarily be in an oxidized form that is incapable of redox functions but may still be competent to modulate non‐redox TRX activities such as binding to certain proteins and transcription factors. ORP100S, when delivered exogenously in the reduced form, will likely be exposed to numerous suitable protein disulfide targets prior to reaching the cell surface. Reaction with these targets will result in binding via thiol‐trapping which will markedly reduce the amount of reduced ORP100S available for cell entry. In contrast, oxidized thiol species and homodimers of ORP100S that are non‐redox active and incapable of thiol‐trapping would have no impediment to uptake. Because the TR/NADPH system is unable to reduce oxidized ORP100S intracellularly these proteins would remain redox‐inactive but could still exert modulatory effects on targets like KLF4 if such regulation proceeded via non‐redox pathways. In our studies we found that unless care is taken to synthesize uniformly reduced C35S TRX and maintain them in the reduced form the oxidized fraction can comprise a significant portion of the total. This was found to be especially important for single C35S mutant ORP‐100 that lacks the additional stabilizing modifications in ORP100S. However, our results show that ORP100S is still as or more potent than natural rhTRX for mediating extracellularly‐directed stress protective functions. We therefore hypothesize that both intracellular and extracellular redox signaling may be required for the protective/proliferative functions of TRX in metabolically‐intensive cancer cells, whereas slower‐metabolizing cells such as EML and cord blood stem cells require only extracellular TRX functions and non‐redox intracellular mechanisms (as could be exerted by oxidized ORP100S) to survive cellular stress and proliferate. Thus, the attenuated intracellular signaling ability of ORP100S may be insufficient to enable stress protection in tumors even with extended extracellular target engagement and enhanced PK/PD.

### 3.5 Human Translation Consideration

Although substantial differences exist between murine models and human disease, TRX is highly conserved at the amino acid level between human and mouse with complete sequence identity in functionally important protein regions. NHP are even more highly conserved with only 2/105 amino acid differences (98% identity). While NHP studies often provide the most clinically relevant preclinical data, several factors require consideration for human translation: 1) allometric scaling suggests a human dose of 10–15 mg m^−2^ based on our minimum effective NHP dose of 7.1 mg m^−2^, which will require safety confirmation in Phase I studies (and the use of higher doses may be supported); 2) species differences in TRX expression levels, reduction status, and cellular uptake mechanisms may affect efficacy; 3) differences in radiation sensitivity between species may require dose adjustments. Development under the FDA Animal Rule pathway requires demonstration of a well‐understood mechanism of action across species, appropriate animal models with endpoints relevant to human survival, and evidence that animal efficacy endpoints are reasonably likely to predict clinical benefit. Our data across multiple species and mechanistic understanding support this regulatory pathway.

### 3.6 Additional Considerations

An interesting aspect of thioredoxin regulation is the potential interaction of ORP100S with the alpha‐arrestin family protein TXNIP (thioredoxin interacting protein), an endogenous TRX inhibitor that acts via trapping of the active‐site Cys thiol. While it is possible that TXNIP could bind reduced ORP100S via its active‐site cysteine and thereby diminish stability or activity, based on our pharmacokinetic studies in NHP we expect that the concentration of SC administered ORP100S will exceed 1000 ng mL^−1^ at Cmax. Normal TXNIP levels in patient serum are in the 30 ng mL^−1^ range.^[^ ^86^ ^]^ Consequently, there will likely be a significant molar excess of ORP100S versus TXNIP and any binding would not substantially affect the available concentration of drug. In fact, we believe that sequestration of TXNIP by binding to excess ORP100S in restricted compartments such as the eye could result in enhanced levels of endogenous active TRX.

Another functional difference between native TRX and ORP100S is the ability of nitric oxide (NO) to modulate certain redox regulatory and anti‐apoptotic functions of TRX in endothelial cells as well as the ability of native TRX through its dithiol active site to act as a regulatory trans‐nitrosylase. In contrast, monothiol active site ORP100S is refractory to S‐nitrosylation by modification of its Cys target site rendering ORP100S insensitive to functional modulation by NO. ORP100S treatment is thus very unlikely to induce crosstalk with normal NO signaling as might occur with delivery of rhTRX.

Chemotherapy‐resistant cancer cells exhibit altered redox homeostasis compared to sensitive cells.^[^ ^87^ ^]^ In particular, resistant lines typically upregulate NRF2‐driven antioxidant defenses to buffer inherently high ROS levels.^[^ ^88^ ^]^ In our study, the activity of ORP100S, a redox active monothiol TRX variant, is unlikely to have intracellular redox activity as described above and hence might not contribute to ROS defense even in resistant cancers. Our study did not explicitly compare isogenic sensitive versus resistant lines, which could be a future direction. However, based on our results and the lack of activity in p53 mutant cells it is likely that ORP100S would not confer further protection to chemo‐resistant cancer cells. In fact, if a cancer cell evolves resistance by elevating TRX/TR expression, an exogenous TRX mutant like ORP100S that lacks intracellular redox function would provide little to no added survival.

### 3.7 ORP100S Therapeutic Development

The robust preclinical efficacy of ORP100S in both small (mouse) and large (primate) animal models and in vitro in multiple murine and human solid tumor and hematopoietic cancer cell lines supports entry into clinical development to evaluate safety, tolerability, and pharmacokinetics in healthy volunteers for radiation mitigation or in patients receiving chemotherapy. If substantiated by efficacy in humanized patient‐derived tumor models, future clinical trial in cancer patients could test if ORP100S administration alongside high‐dose chemotherapy or therapeutic radiation improves treatment outcomes by differentially protecting normal cells, allowing for more rapid and effective tumor shrinkage, blood cell count recovery, or avoidance of neutropenia. Approval as a radiation countermeasure by the US FDA proceeds by the Animal Rule mechanism which is designed to enable licensure of drugs or biologics when clinical efficacy studies are unethical or unfeasible. This regulatory pathway requires only human phase 1 clinical safety studies, whereas other indications proceed via a standard FDA process involving multiple clinical phases to address safety, efficacy and optimal dose in statistically‐relevant population sizes. Filing of an Investigational New Drug (IND) application for any of the potential ORP100S indications would be supported by regulated 28‐day toxicology studies and detailed manufacturing, formulation and analytical data to meet FDA chemistry, manufacturing and controls (CMC) requirements. Immunogenicity and long‐term chronic toxicology studies are typically conducted concurrently with human clinical studies as immunogenicity of human proteins in animals is generally expected to occur, and is hence not informative clinically.

In summary, we describe for the first time an engineered derivative of the potent cellular stress‐protective protein TRX that has properties suitable for development as a safe and effective human therapeutic, and demonstrate that ORP100S both mitigates radiation‐induced injury in vitro and prolongs survival in mice and primates in vivo following irradiation that is uniformly lethal in untreated control animals. ORP100S is also able to protect mouse and human HSPCs from chemotherapy‐induced injury without affecting the efficacy of chemotherapeutic agents against multiple tumor cell types. An important next step is to evaluate the ability of ORP100S to improve cancer treatment efficacy in humanized models or patient‐derived xenografts. ORP100S can be manufactured scalably in *E. coli* and we have developed a highly purified formulation that both enables long‐term storage stability in a fully active, reduced form and that is compatible with multiple routes of administration as a reconstituted solution in normal saline. Both chemoprotection and radiation mitigation in animals and non‐cancer cells treated with ORP100S appear to be mediated in part by enhanced suppression of p53 expression‐mediated ferroptosis. The loss of this protective function in cancer cells treated with ORP100S versus natural thioredoxin likely reflects the existence of multiple TRX‐catalyzed stress‐protective and proliferative signaling pathways, some of which (likely those involving intracellular TRX redox cycling, which is inhibited in ORP100S) are dispensable in non‐cancer cells. These findings provide a strong rationale for therapeutic development of ORP100S.

## 4 Experimental Section

### Animal/Clinical Ethics

All studies involving vertebrate animal models were conducted in accordance with Duke University Institutional Animal Care and Use Committee (IACUC) approved procedures under the following protocol: A073‐23‐03. All studies involving NHPs were performed by Wake Forest University School of Medicine IACUC‐approved procedures under the protocol: A23‐031. De‐identified human nasal airway epithelial cells from normal healthy volunteers or CF subjects were collected under Institutional Review Board (IRB)‐approved protocols (National Jewish Health, Denver CO).

### ORP‐100/ORP100S Design and Manufacturing

The sequence of ORP‐100 (C35S TRX) and ORP100S (C35S TRX plus three additional site‐directed modifications) was codon‐optimized for expression in BL21 *E. coli* using a custom algorithm based on the amino acid sequence of human TRX and cloned into expression vector pD861 (DNA2.0/Atum, Newark CA) under control of a sugar‐inducible promoter. Inducible expression was verified by SDS‐PAGE following growth at small scale in 2 mL volume culture blocks. For 500 g scale manufacturing cells were cultured on glycerol in a 150 L fed‐batch fermenter (105 L working volume; National Research Council of Canada, Montreal) and recombinant expression was induced at appropriate cell density. The fermentation broth was harvested 48 h post‐induction. ORP100S retained intracellularly in soluble form was recovered by cell disruption and heat shock followed by flocculation and depth filtration. Purification of ORP100S from the clarified lysate was performed by anion exchange chromatography in bind‐and‐elute mode, followed by flow‐through anion exchange to remove endotoxins and a hydrophobic interaction chromatography (HIC) polishing step. Dithiothreitol (DTT, Sigma, St. Louis MO) was maintained at 10 mM throughout the downstream processing to ensure that ORP100S remained in the reduced form. Following ultrafiltration/diafiltration (UF/DF) to remove DTT, ORP100S was exchanged into lyophilization buffer at a concentration of 60 to 70 g L^−1^ and frozen (−80 °C). Post anion‐exchange process yields exceeded 70%. The frozen material was subject to lyophilization (Pace Analytical, San Diego CA) to yield the final drug product as a low‐moisture, crystalline white solid. ORP100S was stored long‐term at −80 °C under nitrogen gas to minimize oxidation; reduction state of the lyophilizate remained greater than 90% after more than four years frozen storage.

### ORP‐100/ORP100S Characterization

Size of the expected product and presence of dimers or higher‐order multimers was determined by SDS‐PAGE followed by Coomassie staining. Identity was determined by matrix‐assisted laser desorption/ionization‐time of flight and electropsray ionization mass spectrometry analysis (The Scripps Research Institute, La Jolla, CA). The only post‐translational modification observed was presence of N‐terminal Met. Purity was greater than 99% by SEC‐UPLC.

Reduction state of ORP100S was quantified using 5,5'‐Dithiobis(2‐nitrobenzoic acid (DTNB, Fisher Scientific, Waltham MA) which reacts with free SH groups resulting in a yellow color change at 412 nm. ORP100S concentration was determined by A_280_ (Nanodrop, Fisher Scientific) using the molar extinction coefficient of human Trx‐1 (7,000 M^-1^ cm^-1^). The actual concentrations of free sulfhydryl groups were calculated based on the absorbance at 412 nm and the extinction coefficient of DTNB (14,150 M^-1^ cm^-1^) in order to determine the reduction state as percentage of free sulfhydryl.

Percent of free monomer ORP100S in solution was determined by SEC on an Agilent 1100 HPLC system (Santa Clara, CA) with a BioBasic S‐300 250 × 4.6 column (Fisher Scientific). The ORP100S monomer percentage was determined by integration of the area under the peak of the monomeric fraction divided by the total area under the curve of the A_280_ nm chromatogram.

Protein disulfide bond reduction activity was determined by single‐turnover insulin reduction. rhTRX, ORP‐100, or ORP100S samples were incubated with 10 mg mL^−1^ insulin for various time points (0 – 90 min) and reactions were stopped with iodacetic acid and trifluoroacetic acid. The reaction mixtures were analyzed over RP‐HPLC (Agilent 1100, Santa Clara CA) using an Intrada WP‐RP 50 × 3 column (Imtakt, Portland OR). Relative activity was determined from decrease over time of the insulin heterodimer peak area.

### Primary Bronchial Epithelial Cultures and Cytokine Analysis

Nasal airway epithelial cells from multiple normal healthy volunteers or CF subjects were cultured in serum‐free media at an air‐liquid interface (ALI) with mucociliary differentiation, based on methods adapted from the approach of Schlegel and colleagues.^[^ ^89^ ^]^ 30 wells were cultured to differentiation at ALI from each of three unique normal or CF donors (homozygous for F508del). Triplicate primary cultures were exposed at the apical surface to PBS, native TRX, or ORP‐100 and both apical and basolateral media samples were collected at 4 and 24 h after challenge. Media was centrifuged to remove debris and stored at ‐80 °C until cytokine analysis (ELISA).

### Human Cord Blood Units

Human umbilical cord blood units were obtained from the Carolinas Cord Blood Bank under Duke Institutional Review Board exempt protocol. These cord blood units were not eligible for banking and were provided to qualified investigators for research purpose. Human CD34+ cells were isolated from cord blood using EasySep Human Cord Blood CD34 Positive Selection kit II (17896, STEMCELL Technologies). Primary human CD34+ cells were cultured in IMDM containing 10% fetal bovine serum (FBS) with 100 ng mL^−1^ of human stem cell factor (SCF) (cat. no. 78062.1), 100 ng mL^−1^ human FLT3 ligand (cat. no. 78137), and 100 ng mL^−1^ of human thrombopoietin (cat. no. 78210) from Stemcell Technologies (Vancouver, Canada).

### Cell Lines

EML (CRL‐11691), TRAMP (CRL‐2731), HT29 (HTB‐38), B16‐F10 (CRL‐6475), MM1.R (CRL‐2975), MV4‐11 (CRL‐9591), SW48 (CCL‐231), LS1034 (CRL‐2158), and EG7 (CRL‐2113) cell lines were acquired from American Type Culture Collection (ATCC, Manassas VA). Short tandem‐repeat profiling was performed for cell line authentication. EML cells were cultured in RPMI1640 supplemented with 2 mm GlutaMAX, 10% FBS, and 1% penicillin‐streptomycin. TRAMP, HT29, and B16‐F10 cells were cultured in DMEM with 2 mm GlutaMAX, 10% FBS, and 1% penicillin‐streptomycin.

### Antibodies and Reagents

The following antibodies were used: p53 rabbit polyclonal antibody (cat no. ab131442, Abcam Limited, Cambridge UK), TXN1 polyclonal antibody (cat no. PA587014, ThermoFisher, Waltham MA), MDM2 antibody (cat no. sc965, Santa Cruz Biotechnology, Dallas TX), p21 antibody (cat no. sc6246, Santa Cruz), SLC7A11 polyclonal antibody (cat no. PA5‐116134, Invitrogen, Waltham MA), GPX4 antibody (cat no. 67763‐1‐1 g, proteintech, Rosemont IL), KLF4 antibody (cat no. A6640, ABclonal, Woburn, MA), mouse lineage anbibody (cat no. 561317, BD Pharmingen, San Diego CA), c‐Kit (CD117) antibody (cat no. 558163, BD Biosciences, Franklin Lakes NJ), CD150 antibody (SLAM) (cat no. 115910, BD Biosciences), CD48 antibody (cat no. 103404, BD Biosciences), ASPP1 antibody (cat no. sc‐53903, Santa Cruz), c‐Myc antibody (cat no. 3987S, Cell Signaling Technology, Danvers MA), and GAPDH antibody (cat no. 2118, Cell Signaling Technology). The following reagents were purchased: recombinant human TRX (cat no. 1970‐TX, Lot MGR0323011, RD, Minneapolis MN), Erastin (cat no. S7242, Selleckchem, Hoouston TX), 5‐FU (cat no. F6627, Sigma–Aldrich, St. Louis MO), cisplatin (cat no. PHR1624, Sigma–Aldrich), doxorubicin (cat no. D1515, Sigma–Aldrich), and etoposide (cat no. E1383, Sigma–Aldrich).

### Cell Viability

For the MTT cell viability assay, 5 × 10^4^ cells each were plated in triplicate wells of a 96‐well plate at a final volume of 100 µL containing various concentrations of ORP100S and rhTRX. The cells were cultured at 37 °C in a 5% CO_2_ incubator for various durations. Then, 20 µL of the combined MTS/PMS solution (equivalent to 5 mg mL^−1^ MTT) (cat no. G5421, Promega, Madison WI) was added to each well of the 96‐well assay plate, the plate was incubated for 3–4 h at 37 °C in a 5% CO_2_ incubator and the absorbance at 490 nm was measured using an ELISA plate reader (VERSAmax, Molecular Devices, San Jose, CA).^[^ ^90^ ^]^

### Western Blotting

Cells were harvested, washed with PBS, and re‐suspended in lysis buffer containing 50 mm Tris‐HCl pH 7.4, 150 mm NaCl, 1 mm EDTA, 1% Triton × 100, 1% sodium deoxycholate, and 0.1% SDS (modified 4x Laemmli Buffer, BioRad). The cells were further lysed by brief sonication. The lysates were centrifuged at high speed for 10 min to remove cell debris. Total protein was quantified using a DC protein estimation kit (BioRad) with bovine serum albumin (BSA) standard. Proteins (20 µg per well) were separated by SDS‐PAGE, transferred onto a nitrocellulose membrane, blocked with commercial blocking buffer (Prometheus, Genesee Scientific, San Diego CA) and washed in Tris‐buffered saline containing 0.1% Tween 20 (TBST). Primary antibodies were incubated in commercial blocking buffer at 4 °C overnight. After washing, membranes were incubated with secondary fluorescent anti‐mouse and anti‐rabbit antibodies (IRDye, Licor). Western blot images were acquired on an Odyssey CLX fluorescent imager (LiCor). Quantitative western blot densitometry was performed using ImageJ.^[^ ^91^ ^]^

### Immunofluorescence Confocal Microscopy

Cells were placed on slides using cytospin and fixed using 4% formaldehyde in PBS for 15 min at room temperature. After fixation, slides were blocked with 10% FBS in cell culture medium and incubated overnight at 4 °C with SLC7A11 and GPX4 antibodies. The slides were then washed three times with PBS and stained with Alexa Fluor 594 goat anti‐rabbit antibody (cat. No. R37117, Thermo Fisher Scientific) for 1 h. After three washings with PBS, slides were stained with DAPI (Cell signaling, 4083) for 5 min and mounted with an antifade mounting medium (VECTASHIELD H‐1000, Vector Laboratories, Newark CA). Images were acquired using a confocal laser scanning microscope (SP5 inverted confocal, Leica Microsystems, Deerfield IL). Sequential scanning of different channels was performed at a resolution of 512 × 512 pixels (HC PLAPO CS2 63×1.1 oil objective, Leica).

### Reactive Oxygen Species (ROS) Determination

Peripheral blood mononuclear cells (PBMCs) were isolated from blood samples collected at the indicated time points and ROS levels were measured using 6‐carboxy‐2′,7′‐dichlorodihydrofluorescein diacetate (DCFH‐DA). Briefly, PBMCs were washed with PBS buffer and centrifuged at 400 g for 5 min. 5 × 10^5^ cells were incubated with 10 uM carboxy‐H2DCFDA (Invitrogen) at 37 °C in 5% CO_2_ for 15–30 min. The cells were then washed with 2 mL warm PBS, resuspended in 500 µL PBS, and a minimum of 10000 events were acquired with a BD FacsCanto II flow cytometer (BD Biosciences, Franklin Lakes NJ). Data was analyzed with FlowJo (BD Biosciences).

### Co‐Immunoprecipitation (co‐IP)

Co‐IP of endogeneous KLF4 and TRX was performed using a Pierce crosslink magnetic IP/Co‐IP kit (cat. no. 88805, Thermo Scientific) according to the manufacturer's instructions. In brief, cells were first lysed in 1X complete protease inhibitor cocktail (Roche, Mannheim, Germany) and 1X PhosSTOP phosphatase inhibitor cocktail (Roche) in lysis/wash buffer. In order to prepare the immunological complex, 4 µg of either IgG (as a control) or TRX1/TXNantibody (cat. no. A7638, ABclonal) were cross‐linked to Protein A/G magnetic beads (cat. no. 88802, Thermo Scientific), mixed with 1 mg of protein lysate, and then incubated overnight at 4 °C. Following incubation 10 µl of the complex was removed for western blot assays, and the remaining samples were mixed with magnetic beads and incubated at room temperature for 2 h. The magnetic beads were collected, washed, and eluted in an elution buffer. Lastly, the supernatant was used for western blotting.

### Pharmacokinetic Analysis

C57Bl/6 mice were administered a single dose of ORP100S 64 µg or 128 µg via IV tail vein or dorsal SC injection and blood samples were collected at various time points (0, 15 m, 30 m, 1 h, 2 h, 4 h, 8 h, 16 h). For NHP blood samples were collected at 0, 15 m, 30 m, 45 m, 60 m, 2 h, 6 h, 24 h, 8 d, 15 d. ORP100S levels in plasma were determined using hybrid‐immunocapture LC/MS‐MS^[^ ^92^ ^]^ with selective quantification by mass spectrometry of the ORP100S tryptic peptide SMPTFQFFK following immunoprecipitation. Assay linearity was verified from 1 to 2000 ng mL^−1^ ORP100S in plasma from mouse and NHP. Appropriate dilutions were performed for sample concentrations above the assay upper limit of quantification. PK parameters were calculated using Phoenix WinNonlin (Pharsight Corporation).

### Total Glutathione Determination

The total glutathione (GSH/GSSG) in cell suspensions (5 × 10^6^ cells mL^−1^) was analyzed using a OxiSelect total glutathione (GSSG/GSH) assay kit per the manufacturer's instructions (Cell Biolabs, Inc). The method is based on the reduction of GSSG to GSH by glutathione reductase in the presence of NAPDH and the subsequent addition of chromogen. Chromogen reacts with the thiol group of GSH, with the production of a spectrophotometrically detectable compound at 405 nm. The total glutathione content in the cell suspension was determined using a microplate reader with a glutathione standard calibration curve.

### Ferrous Iron Determination

For intracellular iron measurements, 2 × 10^5^ cells were washed twice in PBS to remove extracellular Fe^2+^ then labeled with 5 µM Bio Tracker far‐red labile Fe^2+^ dye (cat. no. SCT037, EMD Millipore, Burlington MA) by incubation in the dark for 90 min (37 °C, 5% CO_2_) in a tissue culture incubator, washed twice more with PBS, then resuspended in 0.3 mL of PBS. Fluorescence microscopy was carried out with a Cytation 5 multi‐mode reader (Agilent, Santa Clara CA) and intensity was analyzed using ImageJ.

### Lipid Peroxidation Analysis

EML cells were stained with BODIPY C11 lipid probe (Invitrogen) according to the manufacturer's instructions. This BODIPY C11 probe incorporates into membranes, where it undergoes a shift of the fluorescence emission from 590 to 510 nm upon peroxidation by lipid radicals. Briefly, cells were incubated with 5 µm BODIPY 581/911 C11 reagent in PBS at 37 °C for 30 min. Labeled cells were washed and analyzed by flow cytometry (BD FACSCanto II, BD Biosciences). For lipid peroxidation analysis, the peroxidation state of each group was calculated by mean fluorescence intensity (MFI) ratio of the FL1 channel to that of FL3 channel.

### In Vitro Irradiation

EML cells were grown in 25 cm^2^ petri dishes (Corning) in complete IMDM cultured medium (Corning) supplemented with 20% FBS (GeminiBio), 200 ng mL^−1^ mSCF (Fisher Scientific) and 1% penicillin/streptomycin (Sigma). MM1.R, MV4‐11, TRAMP, HT29, EG7, and B16‐F10 cells were grown in similar medium without mSCF. Cells were harvested for use in experiments when they reach confluence, typically 24 h cell passage. Radiation exposure treatments were as described.^[^ ^20^ ^]^

### Mouse Radiation Injury Models

C57BL/6 mice were selected as the primary rodent model due to their well‐characterized hematopoietic system, standardized radiation sensitivity profiles, and extensive use in radiation countermeasure development. This strain provides reproducible dose‐response relationships critical for establishing efficacy parameters. Groups of age‐matched (8–12 weeks old) and sex‐matched C57BL/6 mice were purchased from the Jackson Laboratory (Bar Harbor, ME) or bred in‐house. For radiation exposure experiments, animals were subjected to total body gamma irradiation in a Cs137 Mark II small animal irradiator (JL Shephard) at the specified radiation dosages. Radiation was administered in a single fraction. Following irradiation, animals were administered either ORP100S, recombinant rhTRX, or PBS by tail vein injection (IV) or dorsal injection (SC) beginning 24 h following irradiation and continuing for up to 10 days at the specified dosing frequencies. Animal body weight, activity, and physical health were monitored. For survival experiments, animals were euthanized at humane endpoints or died naturally. Humane endpoints included inability to reach food or water for >12 h, a 20% decrease in baseline weight, lethargy, labored breathing, or inability to remain upright. Peripheral blood was collected at specified time points via maxillary vein puncture. Complete blood counts were obtained on a HemaVet veterinary automated cell counter (Drew Scientific).

### NHP Radiation Injury Model

Cynomolgus macaques were chosen for several critical advantages: 1) closer phylogenetic relationship to humans with similar radiation sensitivity and hematopoietic recovery patterns, 2) body size allowing for clinically relevant dosing and blood sampling schedules, 3) established models recognized by FDA Animal Rule pathway for radiation countermeasure development, and 4) immune system similarities that better predict human responses to therapeutic interventions. The NHP model provides essential bridging data for human dose projections and safety assessments required for regulatory approval. Eight adult female cynomolgous macaques were acquired from a domestic vendor and housed socially in indoor pens at the Wake Forest University Primate Center. All husbandry and studies were conducted under Wake Forest IACUC approved protocols. Irradiation was performed using a Varian 2100 EX dual energy linear accelerator as described previously.^[^ ^44^ ^]^ The single fraction TB dose of 4 Gy was given using 6 MV x rays at a dose rate of 0.69 Gy min^−1^, using a pair of left and right lateral fields at extended distance to deliver one‐half the dose (1 Gy per field) to each animal's sagittal midline. Phantom studies and ionization chamber measurements were used to establish the irradiation geometry and dose delivery parameters. Clinical evaluations of NHPs were conducted daily using a modification of the Children's Clinical Oncology Group toxicity criteria to assess animals for signs of illness. All animals were given parenteral prophylactic antibiotic treatment and supportive symptomatic care. Animals were sedated with ketamine for blood collection (0, 15 m, 30 m, 45 m, 60 m, 2 h, 6 h, 24 h, 8 d, 15 d, 22 d, 29 d, 36 d, 43 d, 50 d, 57 d). Complete blood counts and complete metabolic panel were assayed daily (Antech Diagnostics) for the first 5 d, then 3 times weekly for 30 d, and then weekly for another 30 d.

### Chromatin Immunoprecipitation (ChIP) Assay

ChIP assays were performed according to the manufacturer's protocol (cat. no. 91820, Cell Signaling Technology). Briefly, cells were collected and cross‐linked with 1% formaldehyde. After centrifugation, the resulting pellets were sonicated and the chromatin solution was precleared with 30 µL of ChIP‐grade protein G magnetic beads (cat. no. 9006; Cell Signaling Technology). The soluble fraction was collected, and the chromatin beads were incubated with positive control histone H3 rabbit (cat. no. 4620; Cell Signaling Technology), normal rabbit IgG (cat. no. 2729; Cell Signaling Technology), KLF4 antibdoy (cat. no. AF3640, Bio‐Techne, Minneapolis MN). ChIP‐enriched DNA was analyzed by quantitative PCR using the *TP53* promoter primers as follows: forward CTTCACCTGGATCCTGTGTCTT and the reverse primer GGAAACAGAGGAGGAGACTTCA The enrichment of specific genomic regions was assessed relative to the input DNA, followed by normalization to the respective control IgG values.

### CRISPR‐Cas9 Knockout and Overexpression Plasmid Construction

For generating p53, human KLF4, or mouse klf4 knock out (KO) cells using CRISPR‐Cas 9, single gRNAs targeting the specific gene were cloned in pX330‐U6‐Chimeric_BB‐CBh‐hSpCas9 and lentiCRISPR v1 vectors (Addgene, Watertown MA) as described previously.^[^ ^56^ ^]^ The sgRNA sequences for p53 KO were CTCTCTACAGATGACTGCCA and CCTCGAGCTCCCTCTGAGCC for mouse and human respectively. The sgRNA sequence for human KLF4 KO was AGCGATACTCACGTTATTCGGGG. The sgRNA sequence for mouse klf4 KO was CGCGACACTCACGTTAGTCGGG. Cells were transfected with two PX330 vectors containing two sgRNAs targeting TP53 exons 2–8.

To over‐express KLF4, the human KLF4 cDNA (accession NM_004235) and the mouse klf4 cDNA (accession NM_01 0637) were cloned into the pcDNA3.1 vector (Life Technologies). EML and EG7 cell lines were transfected with either pCDNA3.1 control vector or pCDNA3.1‐KLF4 at 70% confluence in triplicate on 24‐well plates using lipofectamine 3000 transfection reagent (Invitrogen). Briefly, 2 µg of the KLF4 expression plasmid or the control vector was diluted in 125 µg of Opti‐MEM reduced medium (Thermo‐Fisher). Separately, 5 µl of lipofectamine 3000 was diluted in 125 µl of Opti‐MEM. The diluted DNA and lipofectamine solutions were combined and incubated for 5 min at room temperature. The DNA‐lipofectamine complexes were then added dropwise to the cells.

### Construction of Mouse p53 Promoter – Luciferase Reporter

DNA was isolated from mouse cells using the DNeasy kit (Qiagen, Germantown PA). PCR reactions were performed to isolate the promoter region of the p53 gene using the forward primer CTTCACCTGGATCCTGTGTCTT and the reverse primer GGAAACAGAGGAGGAGACTTCA. PCR products were digested with NheI and XbaI and separated on a 1% agarose gel. The 1.2 kb p53 promoter/regulator region was purified using a DNA gel‐band purification kit (cat. no. 28704, Qiagen) and cloned upstream of the firefly luciferase gene in expression reporter vector pGL3 (GenBank accession number U47295, Promega). EML and EG7 cells were grown in 96‐well plate (1×10^4^ cells per well) were transfected with PGL3‐p53 using lipofectamine 3000 (Invitrogen). Cells were harvested 48 h post‐transfection, and luciferase activities were analyzed using a dual‐luciferase reporter assay system (cat. no. E1910, Promega).

### Chemotherapy‐Induced Myelotoxicity Mouse Models

To generate the EG7 lymphoma tumor model, C57BL/6 mice (male and female, 6–8 week old; the Jackson Laboratory) were injected SC with 1 × 10^6^ luciferase‐expressing EG7 cells. When tumors were palpable (>5 mm in diameter), mice were assigned randomly into four groups, receiving an IP injection with PBS buffer, 5‐FU 50 mg kg^−1^ (one dose), ORP100S (128 µg, SC, every other day, five doses), or a combination of 5‐FU and ORP100S. Tumor burden was monitored weekly for a total of three weeks by bioluminescence intensity. Tumor size was determined and the tumor volume (in mm^3^) was calculated using the equation (length × width^2^)/2. Blood was collected weekly from the submandibular vein for CBC analysis. Tumors and bone marrow were harvested at the end of the experiment and used for immunoblotting analysis.

To generate the B16‐F10 melanoma tumor model, C57BL/6 mice (male and female, 6–8 week old; the Jackson Laboratory) were injected SC with 0.5 × 10^6^ B16‐F10 cells. After the tumors became palpable (> 5 mm in diameter), mice were randomly assigned into four groups, receiving an IP injection with PBS buffer, cisplatin 5 mg kg^−1^ (IP, one dose), ORP100S (128 µg, SC every other day, five doses), or a combination of cisplatin and ORP100S. Mice were monitored for three weeks. Tumor size was measured with external calipers every three days, and the tumor volume (in mm^3^) was calculated using the equation (length × width^2^)/2. Blood was collected weekly for CBC analysis. Tumors and bone marrow were harvested at the end of the experiment and used for immunoblotting analysis.

To determine the combinatorial effects of ORP100S and GM‐CSF, C57BL/6 mice (male and female, 6–8 week old; the Jackson Laboratory) were injected SC with 1 × 10^6^ EG7 cells. After the tumors became palpable (> 5 mm in diameter), the mice were randomly assigned into eight groups, receiving an IP injection with PBS buffer, 5‐FU 50 mg kg^−1^ (one dose), ORP100S (128 µg, SC, every other day for five doses), GM‐CSF (2 µg, SC, daily for five d), a combination of 5‐FU and ORP100S, a combination of 5‐FU and GM‐CSF, a combination of ORP100S and GM‐CSF, and combination of 5‐FU, ORP100S and GM‐CSF. Animals were monitored for three weeks. Tumor burden was quantified weekly by bioluminescence intensity. Tumor size was measured by caliper weekly and tumor volume was calculated using the equation (length × width^2^)/2. Blood was collected weekly for CBC analysis. Tumors and bone marrow were harvested at the end of the experiment and used for immunoblotting analysis. BM long‐term HSPC (Lin‐Scal+C‐Kit+CD150+CD48‐), short‐term HSPC (Lin‐Scal+C‐Kit+CD150‐CD48‐) and multi‐potential progenitor cells (Lin‐Scal+C‐Kit+CD150‐CD48+) were quantitated by flow cytometry (BD FACSCanto II).

### Toxicology and Histology Assessment

C57Bl/6 mice (n = 6 per group per dose) were injected with PBS (vehicle), or 128, 1280, 2560 µg or 5120 µg ORP100S IV or SC in a volume of 200 µl PBS once daily for five days. Mice were weighed daily and monitored for changes in activity or behavior. Hematologic indices were measured at day 6, following which the animals were sacrificed. Hematoxylin and eosin (H&E) staining of the sternum, liver, spleen, kidney, and lung was performed and sections were examined for histology. No ORP100S toxicity was observed even at 5120 µg, equivalent to 80 times the radioprotective dose.

### Statistical Analyses

Data are presented as the mean ± SD unless otherwise specified. Assays were conducted in triplicate. Some experiments represent multiple experiments conducted in technical replicates. Two‐sided comparisons were analyzed with the student's one‐sided *t*‐test. Multiple comparisons were conducted by one‐way ANOVA with multiple comparisons (Tukey's multiple comparisons test). Survival analyses were conducted with the log‐rank (Mantel‐Cox) test. Statistical tests were conducted using GraphPad Prism v9 (Graphpad, Inc). P values less than 0.05 were considered significant. *p < 0.05; **p < 0.01; ***p < 0.001; ****p < 0.0001.

## Conflict of Interest

The authors declare no conflict of interest. OrPro Therapeutics and Duke University are applicants for patents relating to monothiol thioredoxin technology (inventors HM, PH, YK).

## Author Contributions

Study conception and design were undertaken by J.W., P.H., and Y.K. Experiments were performed by J.W., H.M., W.G., D.N., X.W., P.M., S.J., and M.Z. Computational disulfide prediction was conducted by P.H. Statistical analysis and interpretation were performed by J.W. and D.N. The original draft was written by J.W., P.H., and Y.K. Critical revision and editing were undertaken by all authors.

## Institutional Review Board Statement

All studies involving vertebrate animal models were conducted in accordance with the laws of the United States and regulations of the Department of Agriculture. Duke University Institutional Animal Care and Use Committee (IACUC) approved procedures were conducted under the following protocol: A073‐23‐03. All studies involving NHPs were performed by Wake Forest University School of Medicine IACUC‐approved procedures under the protocol: A23‐031. De‐identified human nasal airway epithelial cells from healthy volunteers or CF subjects were collected under Institutional Review Board (IRB)‐approved protocols (National Jewish Health, Denver CO).

## Informed Consent Statement

Not applicable.

## Supporting information



Supporting Information

## Data Availability

The data that support the findings of this study are available from the corresponding author upon reasonable request.
