# Supplementary material for: Optimized Monothiol Thioredoxin Derivative (ORP100S) Protects In Vitro and In Vivo from Radiation and Chemotoxicity Without Promoting Tumor Proliferation
Source: Adv Sci (Weinh). 2025 Sep 11;12(42):e04426. doi: 10.1002/advs.202504426 (PMC12622487; doi:10.1002/advs.202504426)
Supplement: Supplementary file 1 — Supporting Information [file ADVS-12-e04426-s001.docx]

**Optimized monothiol thioredoxin derivative (ORP100S) protects *in vitro* and *in vivo* from radiation and chemotoxicity without promoting tumor proliferation**

Supplementary Figure S1-17

Supplementary Table 1-3


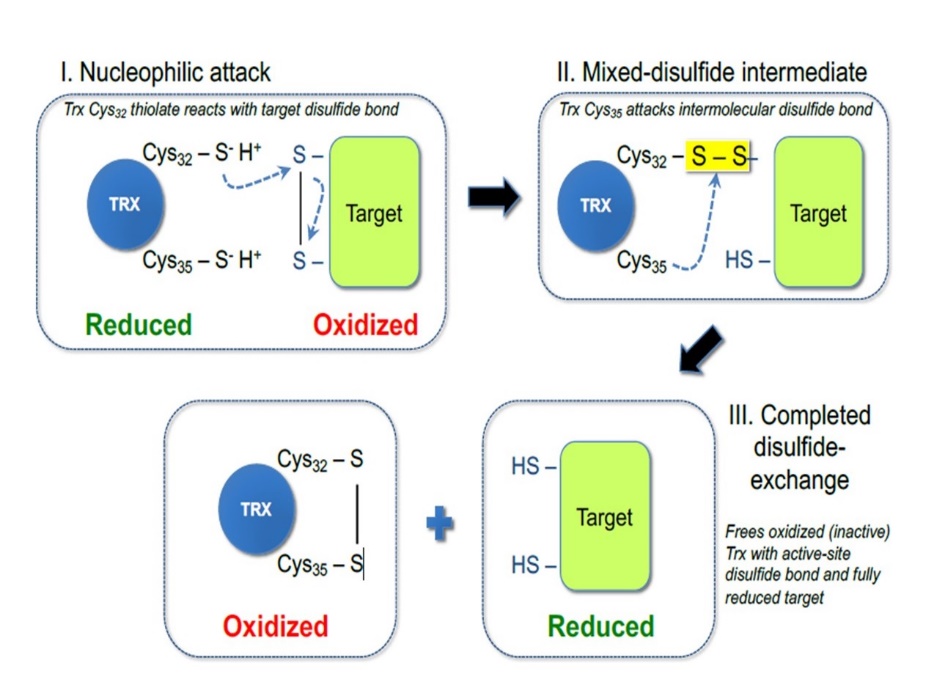


**Supplementary Figure S1.** **Two stage TRX protein-disulfide reduction mechanism.** The N-terminal active-site Cys_32_ thiol forms a reactive thiolate anion when reduced and deprotonated that is capable of nucleophilic attack on a structurally compatible protein disulfide. This initial reaction creates a transition state comprising a mixed-disulfide linkage to one of the two Cys of the target protein disulfide. Formation of this intermediate exposes the normally buried Cys_35_ thiol and displaces onto it the stabilizing active site H-bonds from Cys_32_, causing its thiol pKa to decrease and allowing rapid nucleophilic attack by Cys_35_ on the mixed-disulfide. The products of this second phase are oxidized TRX (with a disulfide bond bridging the two active-site Cys) and a fully reduced protein target. Regeneration of reduced TRX occurs via the thioredoxin reductase (TR) – NADPH system (primarily intracellular) or the glutathione (GSH) – glutaredoxin (GRX) system (primarily extracellular).

**Supplementary Figure S2. Potential binding sites and the 27 possible mixed-disulfide products resulting from reaction of reduced monothiol active site C35S TRX with oxidized heterodimeric insulin (three disulfide bonds).** Alpha (α) and beta (β) denote intermolecular disulfides between insulin A and B chains and gamma (γ) denotes the A chain intramolecular disulfide. Fully oxidized insulin is **α_0_ β_0_ γ_0_**. C35S thioredoxin (ORP100S) will form a mixed-disulfide when it reacts with either Cys of a target protein disulfide bond. Subscripts (1 and 2) denote ORP100S binding to the left side or right side Cys of each Cys-Cys pair, respectively. The table indicates relative abundance (#) of predicted insulin chain – C35STRX adducts (zero, one, two or three bound ORP100S).


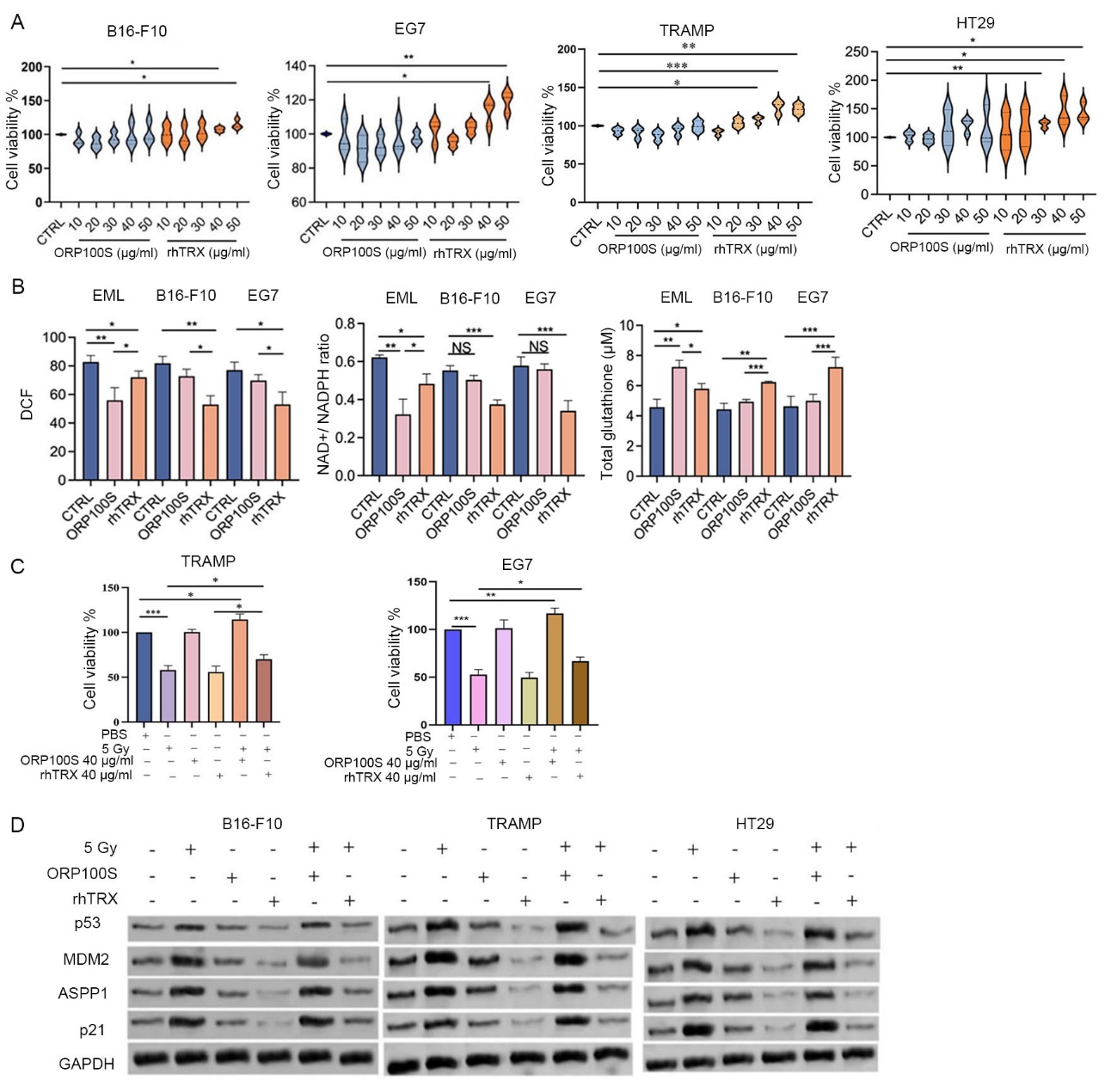


**Supplementary Figure S3.** **ORP100S does not stimulate growth cancer cells growth or rescue cancer cells from radiation. (A)** B16-F10, EG7, TRAMP and HT29 cancer cells were treated with 10 to 50 µg/ml of ORP100S or recombinant human TRX (rhTRX) for 48 hr and cell viability was measured using an MTT colorimetric assay. **(B)** EML, B16-F10 and EG7 cells were treated with ORP100S or rhTRX in PBS (40 µg/ml) for 48 hr and intracellular ROS (DCF), NAD+/NADPH, and total glutathione (GSH) were measured. **(C)** TRAMP and EG7 cancer cells were irradiated (5 Gy) and treated with PBS buffer, ORP100S in PBS (40 µg/ml) or rhTRX in PBS (40 µg/ml) for 48 hr. Cell viabilities were determined by MTT assay. **(D)** Protein lysates from B16-F10, TRAMP and HT29 cells treated as described in **(C)** were subjected to western blotting using the indicated antibodies *: p<0.05, **: p<0.01; ***: p<0.001.


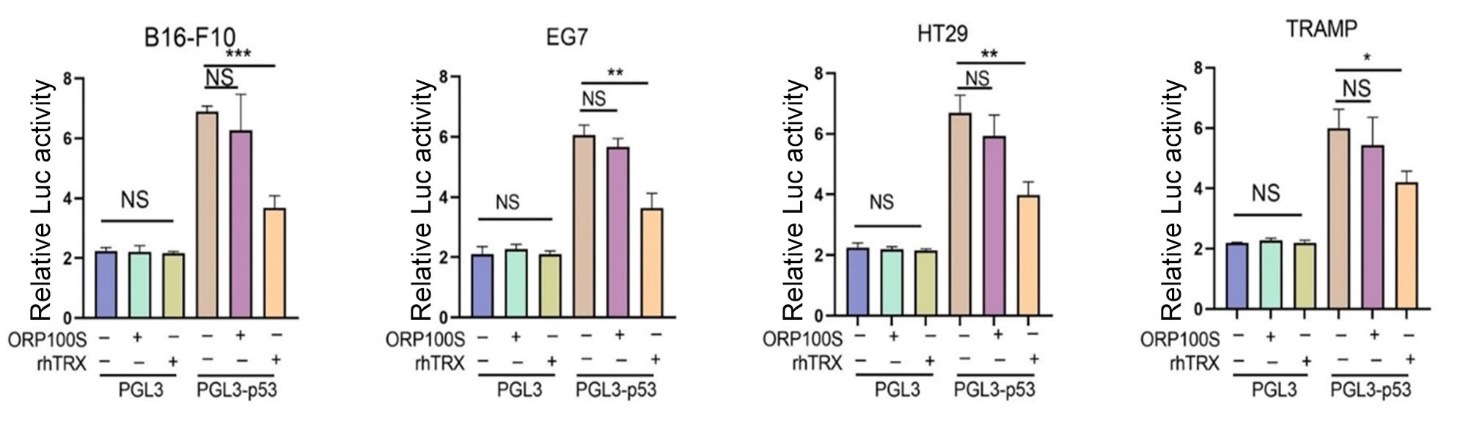


**Supplementary Figure S4. ORP100S does not suppress p53 transcription in cancer cells, unlike rhTRX****.** The promoter region of p53 (-1600 to -100) was cloned into the pGL3 firefly/Renilla luciferase (Luc) reporter system and transduced into four cancer cell lines (B16-F10, EG7, HT29 and TRAMP). Relative Luc activity (fold change) was calculated from the ratio of p53-pGL3 Luc luminescence to pGL3 Luc controls. NS: not statistically significant; *: p<0.05, **: p<0.01; ***: p<0.001.


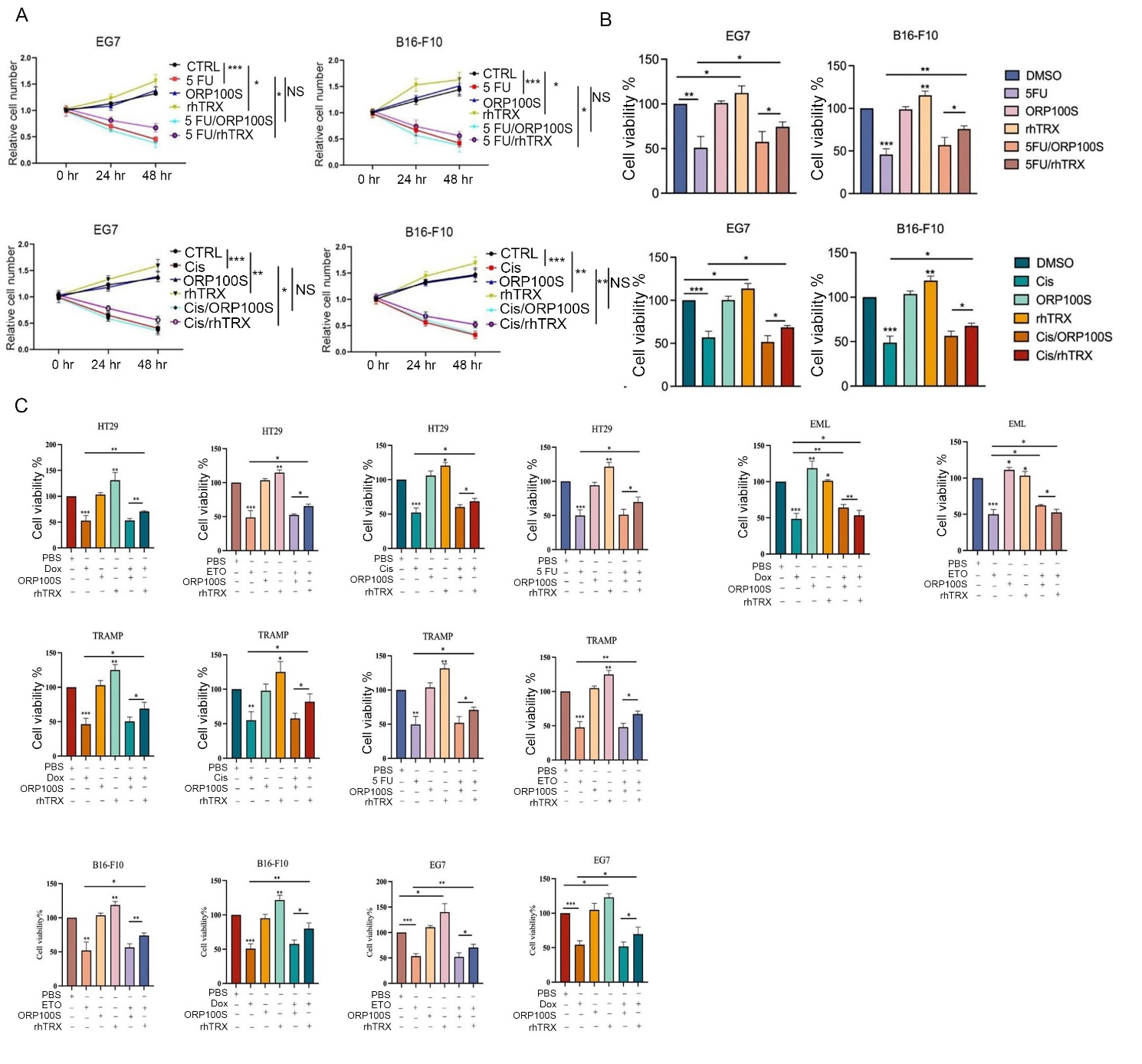


**Supplementary Figure S5. Differential protective and stimulatory effects of ORP100S versus rhTRX in murine hematopoietic stem cells and cancer cells *in vitro*.** **(A)** EG7 and B16-F10 cells were treated with 5-FU (25 μM, upper panel) or cisplatin (Cis, 1 μM, lower panel) with or without ORP100S or rhTRX (40 μg/ml) for 48 hr. Cell counts were determined by Trypan blue staining. Data represent the mean ± SD of three experiments. Cell number at baseline before treatment was normalized to 1.0. **(B)** EG7 and B16-F10 cells were treated with 5-FU (25 μM) or Cis (1 μM) with or without ORP100S or rhTRX (40 μg/ml) and cell proliferation (viability) was determined by MTT assay. **(C)** EML cells were treated with etoposide (ETO, 1 μM) and doxorubicin (Dox, 1 μM) with or without ORP100 (40 μg/ml) or rhTRX (40 μg/ml) for 48 hr. Cell viabilities were determined by MTT assay. HT29 cells were treated with Cis (30 μM), 5-FU (10 μM), ETO (10 μM) and Dox (10 μM) with or without ORP100 (40 μg/ml) or rhTRX (40 μg/ml) for 48 hr. TRAMP cells were treated with Cis (1 0μM), 5-FU (15 μM), ETO (1 μM) and Dox (10 μM) with or without ORP100 (40 μg/ml) or rhTRX (40 μg/ml) for 48 hr. B16-F10 cells were treated with ETO (10 μM) and Dox (10 μM) with or without ORP100 (40 μg/ml) or rhTRX (40 μg/ml) for 48 hr. EG7 cells were treated with ETO (5 μM) and Dox (1 μM) with or without ORP100 (40 μg/ml) or rhTRX (40 μg/ml) for 48 hr. Cell viabilities were determined by MTT assay. Results are presented as mean ±SD from at least three separate experiments. *: p<0.05; **: p<0.01; ***: p<0.001.


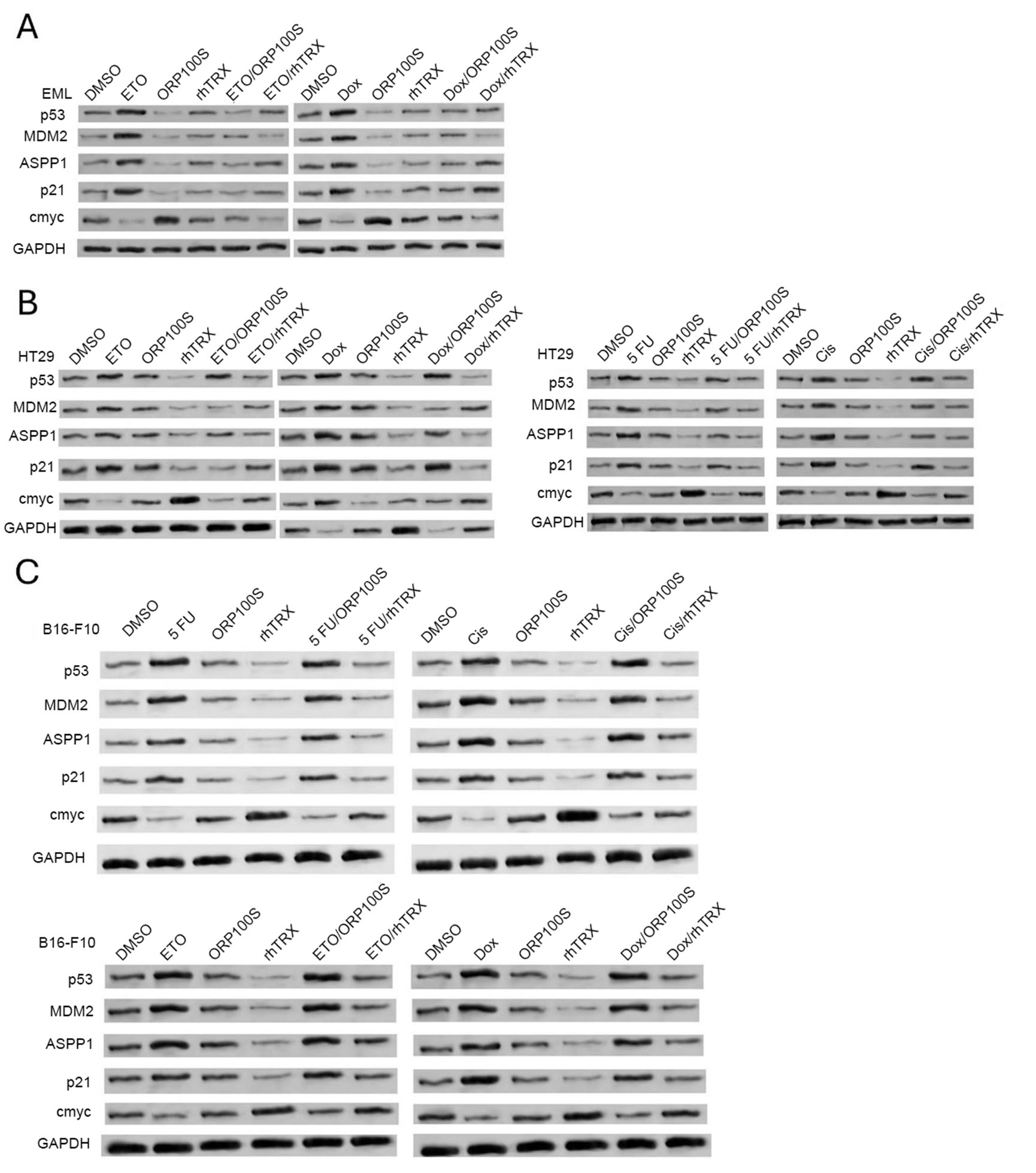


**Supplementary Figure S6. ORP100S attenuates upregulation of the p53 pathway by chemotherapeutic agents in EML cells but not in cancer cells, unlike rhTRX.** **(A)** EML cells were treated with etoposide (ETO, 1 μM) and doxorubicin (Dox, 1 μM) with or without ORP100 (40 μg/ml) or rhTRX (40 μg/ml) for 48 hr. Protein lysates were subjected to Western blotting with indicated antibodies. **(B)** HT29 cells were treated with cisplatin (Cis, 30 μM), 5-FU (10 μM), ETO (10 μM) and Dox (10 μM) with or without ORP100 (40 μg/ml) or rhTRX (40 μg/ml) for 48 hr. Protein lysates were subjected to Western blotting with indicated antibodies. **(C)** B16-F10 cells were treated with 5-FU (25 μM), Cis (1 μM), ETO (10 μM) and Dox (10 μM) with or without ORP100 (40 μg/ml) or rhTRX (40 μg/ml) for 48 hr. Protein lysates were subjected to Western blotting with indicated antibodies.


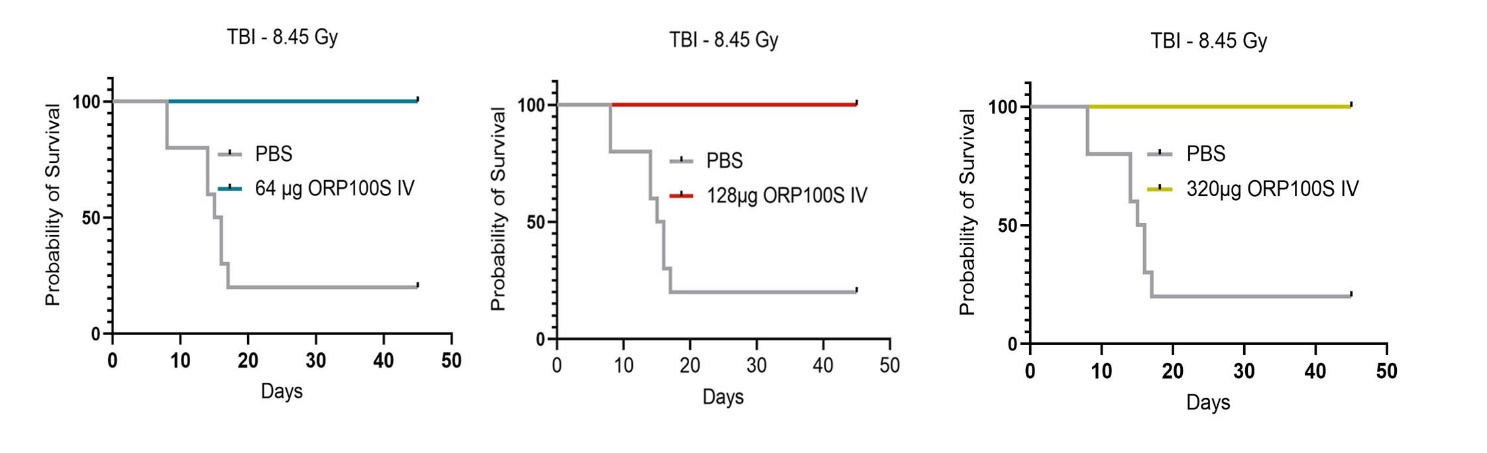


**Supplementary Figure S7.** Survival data from main text Figure 4B separated into individual ORP100S dose vs. control plots for clarity. C57BL/6 mice received 8.45 Gy TBI and 24 hr later were administered PBS buffer or ORP100S (64, 128 and 320 μg, iv) in PBS every other day for five doses. n=10 mice per treatment group.


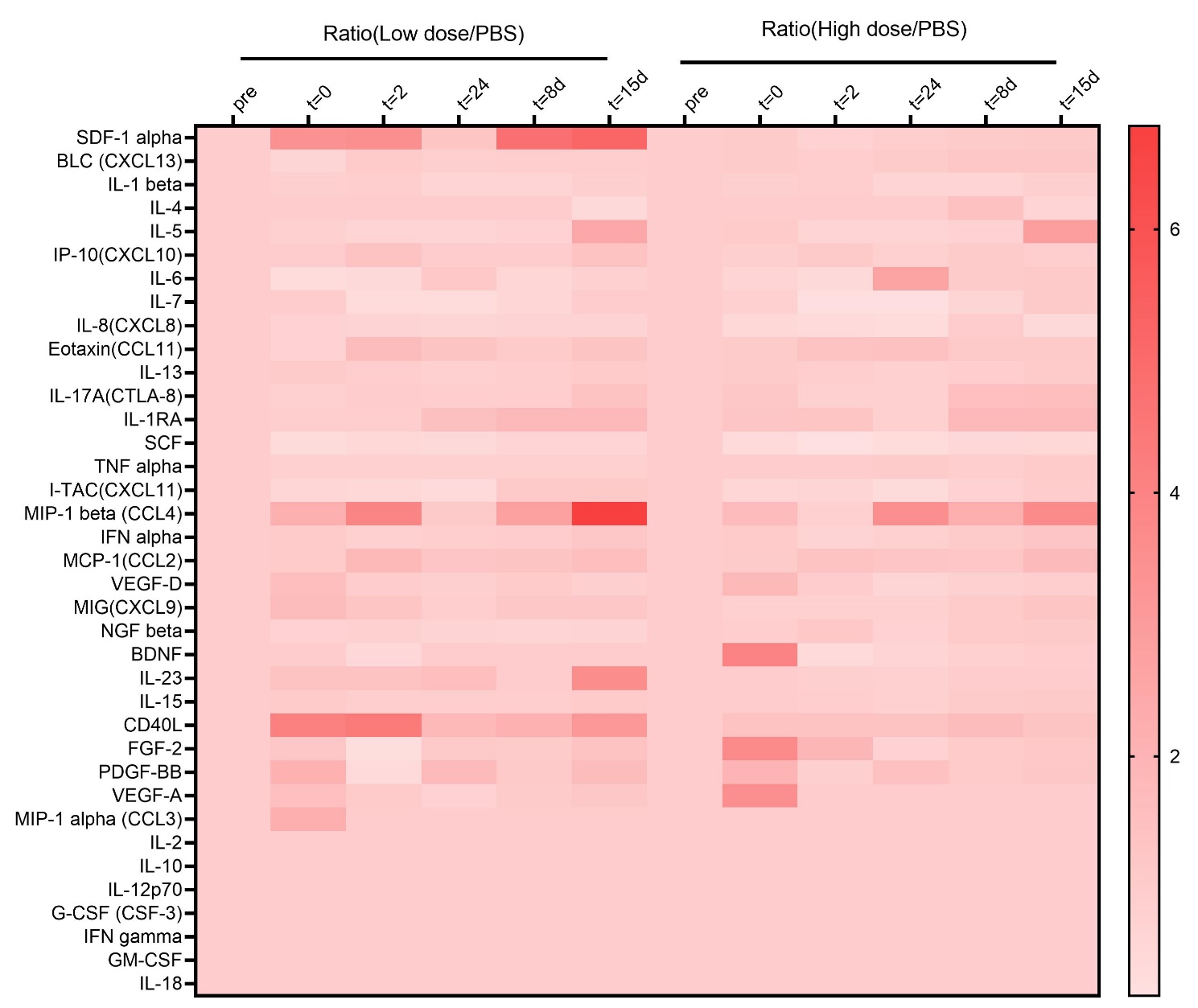


**Supplementary Figure S8.** Cytokines and chemokines from blood samples taken at the indicated time points were measured by Thermo Fisher ProcartaPlex™ NHP 37-plex cytokine/chemokine/growth factor panel (cat. no. EPX370-40045-901). The data shown are the ratios of levels between low dose/PBS-treated and high dose/PBS-treated. *: p<0.05, **: p<0.01; ***: p<0.001.


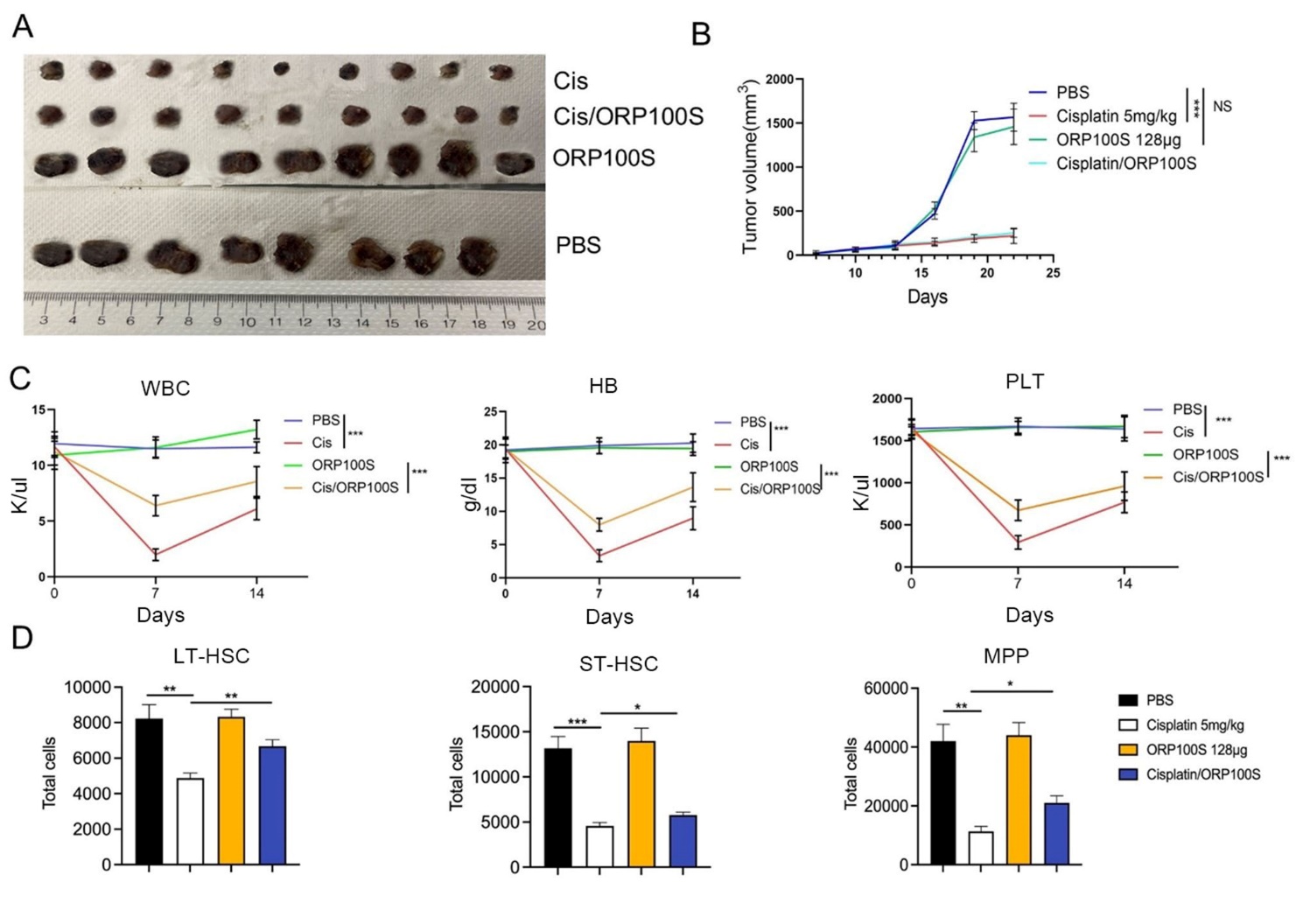


**Supplementary Figure S9. ORP100S protects hematopoietic stem/progenitor cells (HSPC) from chemotherapy-induced hematological toxicity without affecting tumor killing.** C57Bl/6 mice were implanted with B16-F10 melanoma cells and when tumors were established, the mice were given a single dose of PBS vehicle or cisplatin (Cis, 5 mg/kg, IP) followed by treatment with PBS or ORP100S (128 μg, SC every other day for five doses). (**A**) Mice were euthanized, and tumors harvested at two weeks after Cis injection for imaging of tumors from the four groups of mice. (**B**) Tumor volume was measured every three days. (**C**) Blood samples were collected before Cis injection and then at days seven and 14. WBC, HB, and PLT counts were determined over time. (**D**) Total counts from bone marrow of LT-HSC, ST-HSC, and MPP are shown. Data represent means ± SD (n = 10 mice per group) *: p<0.05, **: p,0.01; ***: p<0.001.


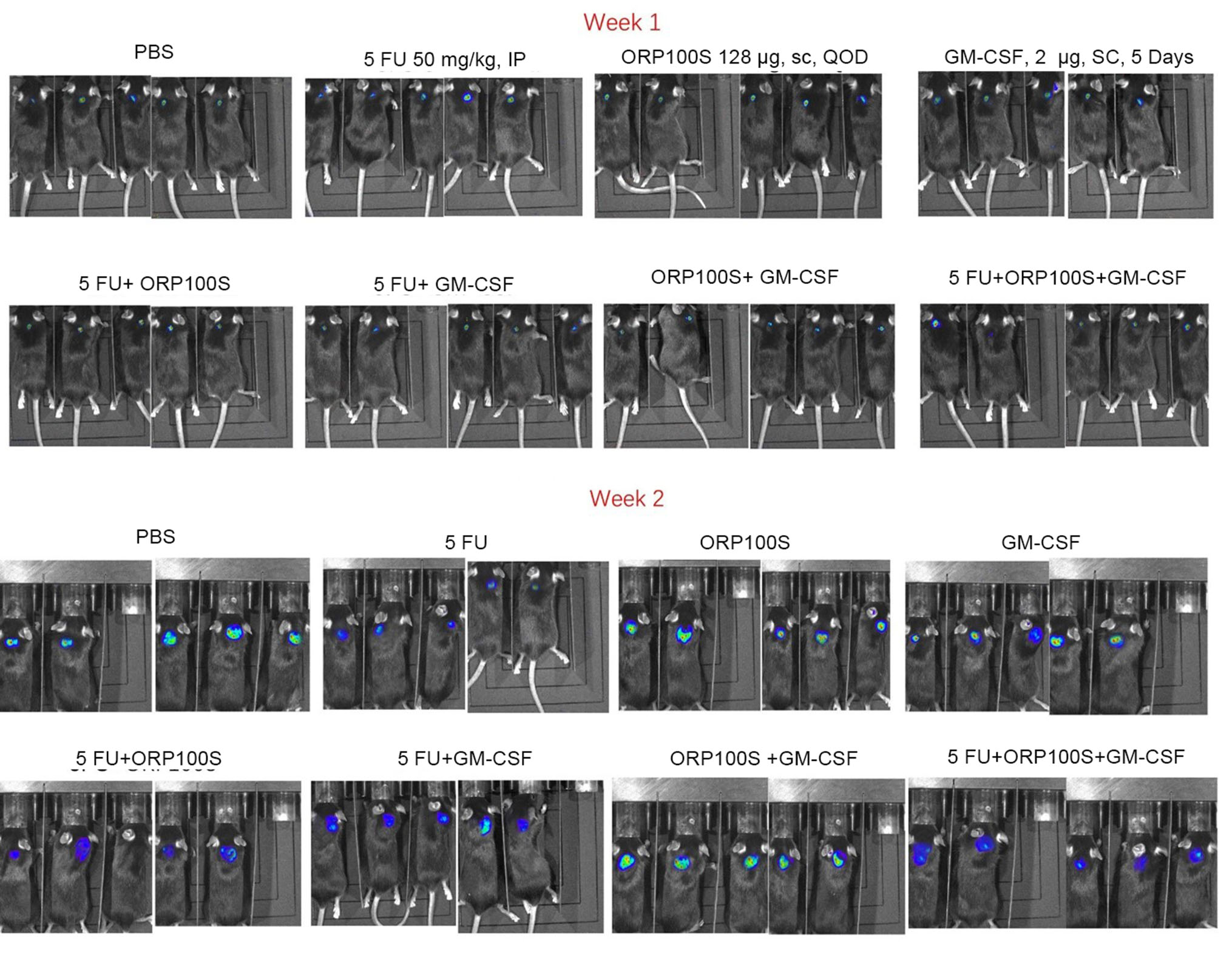


**Supplementary Figure S10. ORP100S and GM-CSF exert additive effects on hematopoietic recovery without affecting 5-FU tumor killing.** C57Bl/6 mice (eight groups, n = 5–6 mice per group) were implanted with luciferase-expressing EG7 cells and treated with PBS or 5-FU intraperitoneally (50 mg/kg, single dose) once tumors were established. Mice were then given PBS control buffer, ORP100S (128 μg, SC every other day for five doses), GM-CSF (2 µg, SC, daily for five days), or a combination of ORP100S and GM-CSF. Tumor volume was determined by bioluminescence imaging at week 1 and week 2.


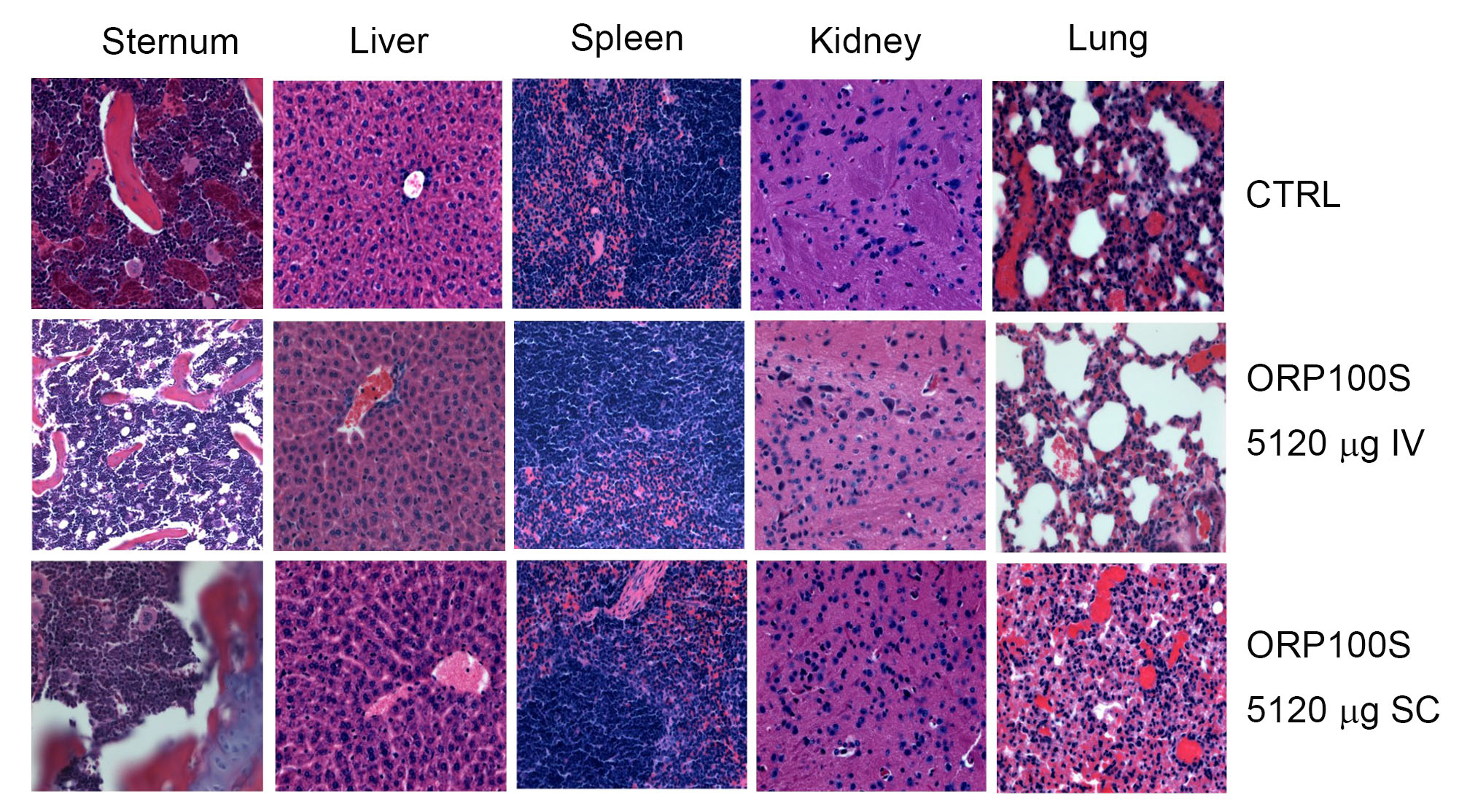


**Supplementary Figure S11**. Representative H&E-stained sections (40× magnification) of sternum (bone marrow), liver, spleen, kidney, and lung from PBS control and ORP100S-treated mice. For each organ, tissue from a PBS control mouse is shown alongside tissue from mice that received a single 5120 µg dose of ORP100S via IV or SC injection. No significant histopathological differences or lesions are evident between ORP100S-treated and control organs.


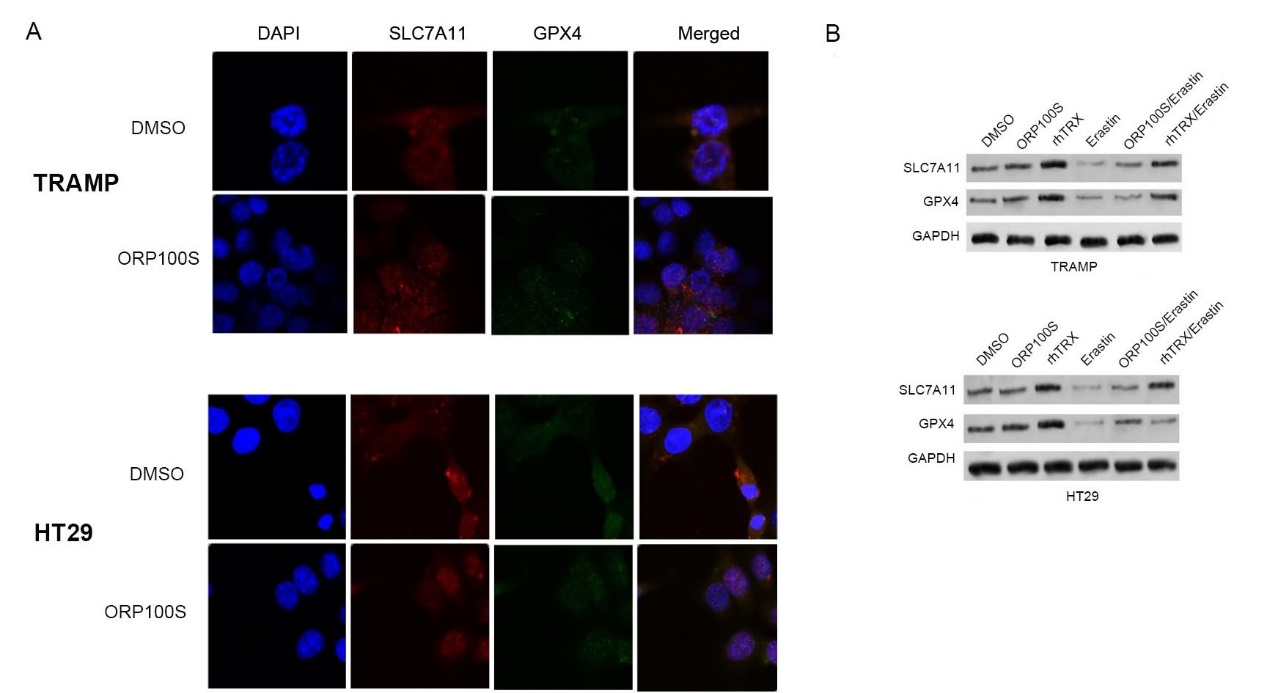


**Supplementary Figure S12. ORP100S but not rhTRX attenuates chemotherapy-induced ferroptosis in EML cells but has no effect on ferroptosis cancer cells. (A)** TRAMP (top) and HT29 cells (bottom) were treated with PBS, ORP100S (40 μg/ml) or rhTRX (40 μg/ml) for 48 hr, following which SLC7A11 and GPX4 were visualized by immunostaining and fluorescence microscopy (40X). (**B)** EML, HT29, TRAMP, and B16-F10 cells were co-treated with Erastin (10µM) +/- ORP100S (40 µg/ml) or rhTRX (40 µg/ml) for 48 hr. SLCA11 and GPX4 were measured by Western blot analysis.


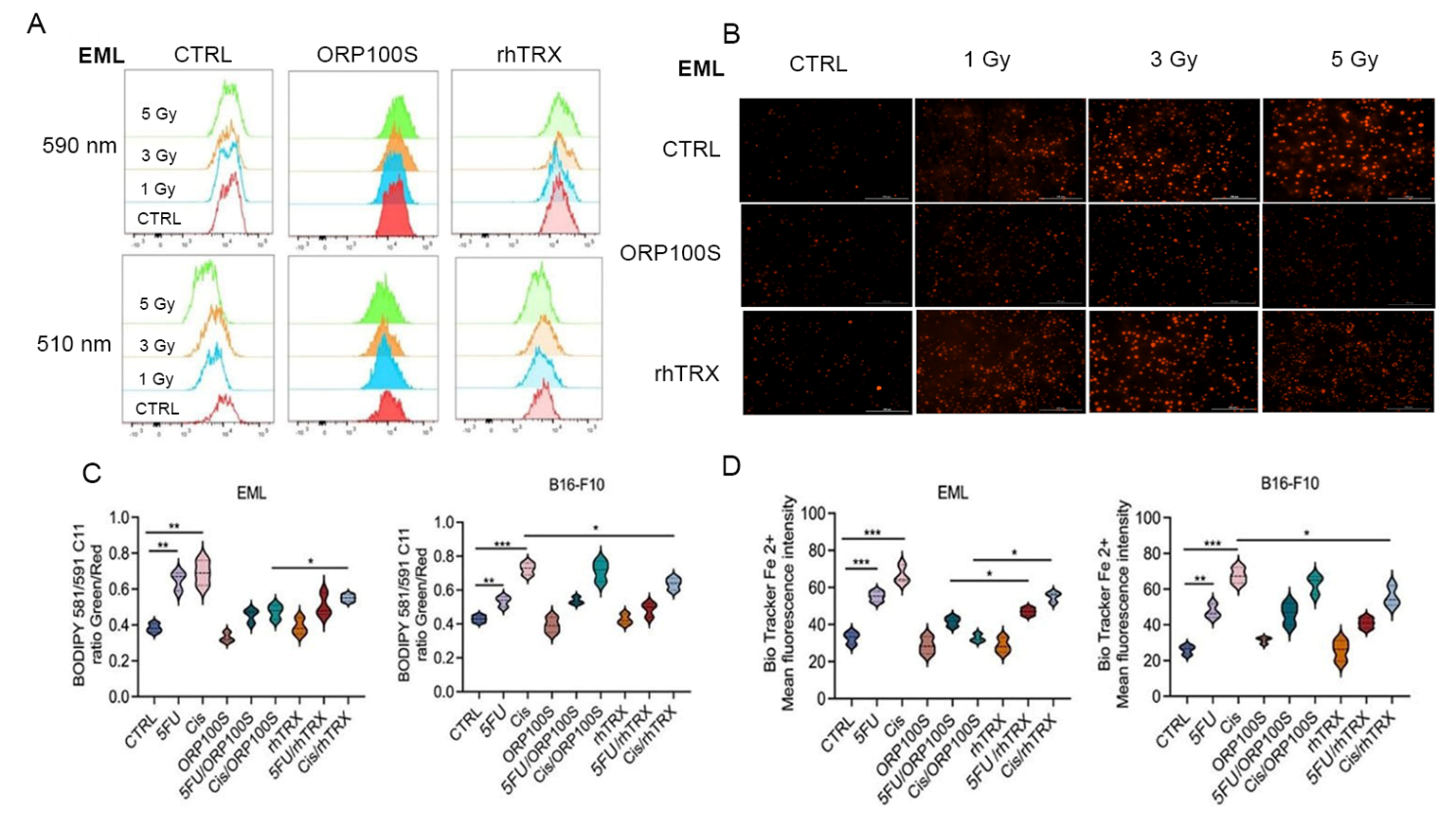


**Supplementary Figure S13. ORP100S treatment suppresses EML cell ferroptosis. (A)** EML cells were irradiated (1 Gy, 3 Gy, or 5 Gy) then immediately treated with ORP100S (40 µg/ml) or TRX (40 µg/ml). Cells were collected after 48 hr and stained with 5 µM BODIPY 581/911 C11 reagent in PBS at 37℃ for 30 min. Labeled cells were washed and analyzed by flow cytometry as described previously. For lipid peroxidation analysis, the peroxidation state of each group was calculated by ratio of the mean fluorescence intensity (MFI) of the FL1 channel (590 nm) to that of FL3 channel (510 nm). **(B)** EML cells were irradiated and treated with ORP100S (40 µg/ml) or TRX (40 µg/ml) as above, collected after 48 hr and labeled with Bio Tracker Far-red Labile Fe2+ Dye 5 µM for 90 min in PBS at 37°C. Representative image analysis was performed using a BioTek Cytation 5 imaging reader. **(C- D)** EML and B16-F10 cells were treated with 5-FU (25 μM) or cisplatin (1 μM) with or without ORP100S (40 μg/ml) or rhTRX (40 μg/ml) for 48 hr. Lipid peroxidation (C) and intracellular ferrous iron levels (D) were measured.


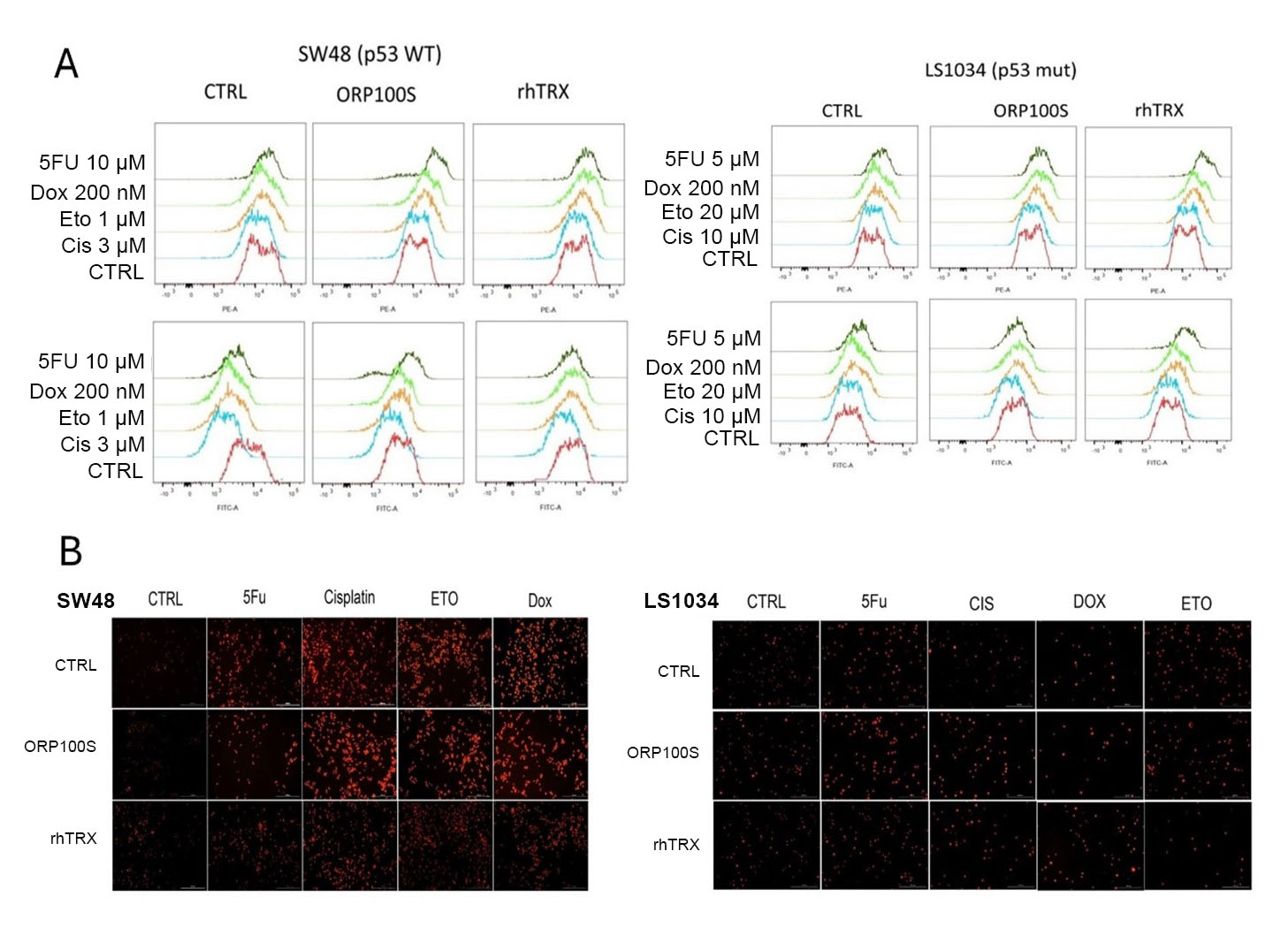


**Supplementary Figure S14. p53 is required for ORP100S-mediated ferroptosis inhibition. (A)** SW48 (p53 WT) and LS1034 (p53 mut) cells were treated with 5-FU, Doxorubicin (Dox), etoposide (Eto) or cisplatin (Cis) at indicated concentration with and without ORP100S (40 μg/ml) or rhTRX (40 μg/ml) in PBS (CTRL). Cells were collected after 48 hr incubation and stained with 5 µM BODIPY 581/911 C11 reagent in PBS at 37℃ for 30 min. Labeled cells were washed and analyzed by flow cytometry. For lipid peroxidation analysis, the peroxidation state of each group was calculated by ratio of mean fluorescence intensity (MFI) of the FL1 channel (590 nm) to that of FL3 channel (510 nm). (**B**) SW48 (p53 WT) and LS1034 (p53 mut) cells were treated with 5-FU, doxorubicin, etoposide or cisplatin at indicated concentration with and without ORP100S (40 μg/ml) or rhTRX (40 μg/ml) in PBS (CTRL). Cells were collected after 48 hr and labeled with Bio Tracker Far-red Labile Fe2+ Dye 5 µM for 90 min in PBS at 37°C. Image capture was performed using a BioTek Cytation 5 imaging reader.


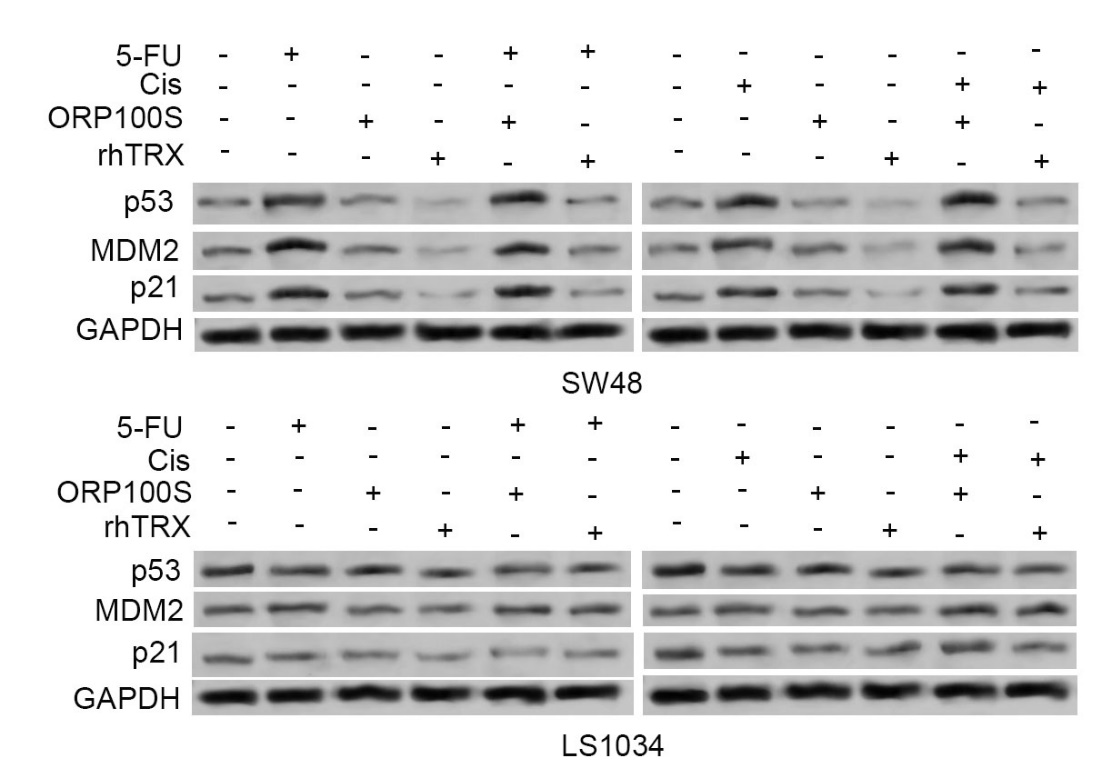


**Supplementary Figure S15. Wildtype but not mutant p53 status is required for TRX-mediated chemoprotection.** SW48 (p53 wildtype) and LS1034 (p53 mutant) cells were treated with cisplatin (30 μM), 5-FU (10 μM), with or without ORP100 (40 μg/ml) or rhTRX (40 μg/ml) for 48 hr. Protein lysates were subjected to Western blotting with indicated antibodies.


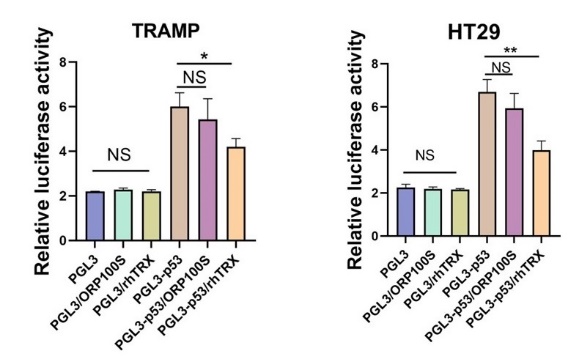


**Supplementary Figure S16**. **ORP100S does not affect p53 transcription in cancer cells.** The p53 promoter region was cloned into the PGL3 firefly/renilla reporter system and the resultant PGL3-p53-reporter plasmid was transduced into EML, B16-F10 and EG7 cells. Transduced cells were then treated with ORP100S (40 μg/ml) or rhTRX (40 μg/ml) for 48 hr, and luciferase bio-luminescence activity was measured.


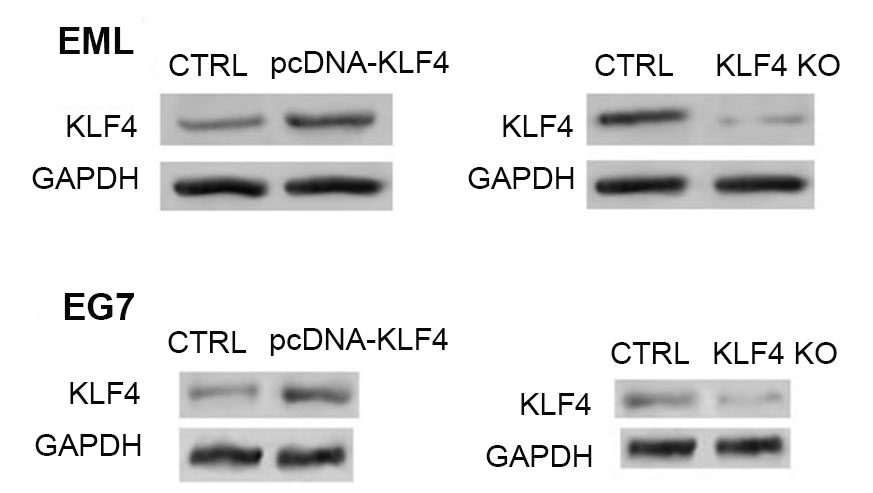


**Supplementary Figure S17. Overexpression or knockout of KLF4 in EML and EG cells.** EML and EG7 cell lines were transfected with control empty vector or pcDNA-KLF4 (left panels) and overexpression of KLF4 after 48 hr was confirmed by Western blot analysis. EML and EG7 cell lines were transfected with control RNA or KLF4-specific sgRNA for CRISPR/Cas9 knock out. Downregulation of KLF4 expression was confirmed after 48 hr by Western blot analysis.

**Supplementary Table 1: Pharmacokinetics of ORP100S in mice**

| **PK Parameter** | **IV-64** | **IV-128** | **SC-64** | **SC-128** |
| --- | --- | --- | --- | --- |
| **tmax** [hr] | 0.0 | 0.0 | 0.5 | 0.5 |
| **Cmax** [ug/mL] | 7.96 | 19.39 | 2.48 | 3.73 |
| **AUC** (area 0-infinity) [ug mL-1 h] | 5.0 | 11.2 | 5.6 | 10.7 |
| **t1/2** (terminal half-life 2-8 h) [h] | 2.09 | 2.54 | 2.51 | 1.91 |
| **t1/2** (terminal half-life 8-16 h) [h] | 4.46 | 5.24 | 3.56 | 2.49 |
| **Cl** (clearance as Dose/AUCINF) [L/h/kg] | 0.64 | 0.57 |  |  |
| **Cl/F** (clearance as Dose/AUCINF) [L/h/kg] |  |  | 0.57 | 0.60 |
| **Vd**(z) (vol term phase) [L/kg] | 4.1 | 4.3 |  |  |
| **Vd/F**(z) (vol term phase) [L/kg] |  |  | 2.9 | 2.2 |
| **Vss** (volume of distribution at steady-state) [L/kg] | 1.6 | 2.0 |  |  |
| **MRT** (mean residence time, 0-infinity) [h] | 2.4 | 3.5 | 3.2 | 2.6 |
| **BIOAVAILABILITY (F)** = AUC(s.c.)/AUC(i.v.) |  |  | **112%** | **95%** |
| **BIOAVAILABILITY (F)** using 2-compartment modeling of IV data |  |  | **108%** | **71%** |

**Supplementary Table 1: Pharmacokinetics analysis of ORP100S in mouse plasma after 64 μg or 128 μg dose administered by intravenous (IV) and subcutaneous (SC) injection.** C57Bl/6 mice were given a single injection of ORP100S 64 µg or 128 µg IV via tail vein or SC via dorsal injection and blood samples were collected for plasma isolation at various time points (0, 15 min, 30 min, 1 hr, 2 hr, 4 hr, 8 hr, 16 hr). ORP100S levels in plasma were determined using hybrid-immunocapture LC/MS-MS with selective quantification by mass spectrometry of the ORP100S tryptic peptide SMPTFQFFK following immunoprecipitation. Plasma PK parameters were calculated using non-compartmental and 2-compartment approaches (WinNonlin).

**Supplementary Table 2: Pharmacokinetics of ORP100S in nonhuman primates (NHPs)**

| **PK Parameter** | **Low Dose** | **stdev** | **High Dose** | **stdev** |
| --- | --- | --- | --- | --- |
| **tmax** [hr] | 6 | 0 | 6 | 0.0 |
| **Cmax** [ug/mL] | 1.09 | 0.07 | 1.83 | 0.30 |
| **AUC** (area 0-infinity) [ug mL-1 h] | 18.4 | 2.2 | 35.5 | 9.8 |
| **t1/2** (terminal half-life 6-24 h) [h] | 6.94 | 1.25 | 8.97 | 1.69 |
| **Cl/F** (clearance as Dose/AUCINF) [L/h/kg] | 0.026 | 0.003 | 0.026 | 0.006 |
| **Vd/F**(z) (vol term phase) [L/kg] | 0.261 | 0.026 | 0.332 | 0.029 |
| **MRT** (mean residence time, 0-infinity) [h] | 7.0 | 0.6 | 7.8 | 0.6 |

**Supplementary Table 2: Pharmacokinetic analysis of ORP100S in NHP plasma after subcutaneous (SC) injection of 7.1 mg/m^2^ (low dose) or 14.2 mg/m^2^ (high dose) ORP100S.** Cynomolgous macaques mice were given a single SC injection of ORP100S (64 µg or 128 µg) and blood samples were collected for plasma isolation at various time points (0, 15 min, 30 min, 45 min, 60 min, 2 hr, 6 hr, 24 hr, 8 d, 15 d). ORP100S levels in plasma were determined using hybrid-immunocapture LC/MS-MS with selective quantification by mass spectrometry of the ORP100S tryptic peptide SMPTFQFFK following immunoprecipitation. Plasma PK parameters were calculated using non-compartmental and 2-compartment approaches (WinNonlin)**.**

**Supplementary Table 3: Comparison of plasma PK parameters between mouse and nonhuman primate (NHP)**

|  | NHP | | MOUSE | | NHP/MOUSE | |
| --- | --- | --- | --- | --- | --- | --- |
| PK Parameter | Low Dose | High Dose | Low Dose | High Dose | Low Dose | High Dose |
| Dose (nominal) [mg/kg] | 0.48 | 0.9 | 3.2 | 6.4 | 0.15 | 0.14 |
| tmax [h] | 6 | 6.0 | 0.5 | 0.5 | 12.00 | 12.00 |
| Cmax [ug/mL] | 1.09 | 1.83 | 2.48 | 3.73 | 0.44 | 0.49 |
| AUC (area 0-infinity) [ug mL-1 h] | 18.4 | 35.5 | 5.6 | 10.7 | 3.27 | 3.33 |
| t1/2 (terminal half-life 8-16 h) [h] | 6.94 | 8.97 | 3.56 | 2.49 | 1.95 | 3.60 |
| Cl/F (clearance as Dose/AUCINF) [L/h/kg] | 0.03 | 0.03 | 0.57 | 0.60 | 0.05 | 0.04 |
| Vd/F(z) (vol term phase) [L/kg] | 0.3 | 0.3 | 2.9 | 2.2 | 0.09 | 0.15 |
| MRT (mean residence time, 0-infinity) [h] | 7.0 | 7.8 | 3.2 | 2.6 | 2.22 | 2.97 |

**Supplementary Table 3. Plasma pharmacokinetics of ORP100S in mouse and NHP after subcutaneous administration.** The ORP100S concentration in plasma was determined using hybrid immunocapture LC/MS-MS. Plasma PK parameters were calculated using non-compartmental and 2-compartment approaches (WinNonlin). Low dose: 64 μg per mouse, equivalent to 7.1 mg/m^2^ in NHP. High dose: 128 μg per mouse, equivalent to 14.2/ m^2^ in NHP.
